# Explaining disparities in oncology health systems delays and stage at diagnosis between men and women in Botswana: A cohort study

**DOI:** 10.1371/journal.pone.0218094

**Published:** 2019-06-06

**Authors:** Hari S. Iyer, Racquel E. Kohler, Doreen Ramogola-Masire, Carolyn Brown, Kesaobaka Molebatsi, Surbhi Grover, Irene Kablay, Memory Bvochora-Nsingo, Jason A. Efstathiou, Shahin Lockman, Neo Tapela, Scott L. Dryden-Peterson

**Affiliations:** 1 Department of Epidemiology, Harvard T. H. Chan School of Public Health, Boston, Massachusetts, United States of America; 2 Department of Social and Behavioral Sciences, Harvard T. H. Chan School of Public Health, Boston, Massachusetts, United States of America; 3 Dana-Farber Cancer Institute, Boston, Massachusetts, United States of America; 4 Botswana University of Pennsylvania Partnership, Gaborone, Botswana; 5 School of Medicine, University of Botswana, Gaborone, Botswana; 6 Department of Obstetrics and Gynecology, Perelman School of Medicine, University of Pennsylvania, Philadelphia, Pennsylvania, United States of America; 7 Department of Epidemiology, Emory Rollins School of Public Health, Atlanta, GA, United States of America; 8 Department of Statistics, University of Botswana, Gaborone, Botswana; 9 Department of Radiation Oncology, Perelman School of Medicine, University of Pennsylvania, Philadelphia, Pennsylvania, United States of America; 10 Princess Marina Hospital, Gaborone, Botswana; 11 Nyangabgwe Hospital, Gaborone, Botswana; 12 Department of Oncology, Gaborone Private Hospital, Gaborone, Botswana; 13 Harvard Medical School, Boston, Massachusetts, United States of America; 14 Division of Infectious Diseases, Department of Medicine, Brigham and Women’s Hospital, Boston, Massachusetts, United States of America; 15 Botswana Harvard AIDS Institute Partnership, Gaborone, Botswana; 16 Botswana Ministry of Health and Wellness, Gaborone, Botswana; 17 Nuffield Department of Population Health, University of Oxford, Oxford, United Kingdom; 18 Department of Immunology and Infectious Diseases, Harvard T. H. Chan School of Public Health, Boston, Massachusetts, United States of America; Duke University, UNITED STATES

## Abstract

**Purpose:**

Men in Botswana present with more advanced cancer than women, leading to poorer outcomes. We sought to explain sex-specific differences in time to and stage at treatment initiation.

**Methods:**

Cancer patients who initiated oncology treatment between October 2010 and June 2017 were recruited at four oncology centers in Botswana. Primary outcomes were time from first visit with cancer symptom to treatment initiation, and advanced cancer (stage III/IV). Sociodemographic and clinical covariates were obtained retrospectively through interviews and medical record review. We used accelerated failure time and logistic models to estimate standardized sex differences in treatment initiation time and risk differences for presentation with advanced stage. Results were stratified by cancer type (breast, cervix, non-Hodgkin’s lymphoma, anogenital, head and neck, esophageal, other).

**Results:**

1886 participants (70% female) were included. After covariate adjustment, men experienced longer excess time from first presentation to treatment initiation (8.4 months) than women (7.0 months) for all cancers combined (1.4 months, 95% CI: 0.30, 2.5). In analysis stratified by cancer type, we only found evidence of a sex disparity (Men: 8.2; Women: 6.8 months) among patients with other, non-common cancers (1.4 months, 95% CI: 0.01, 2.8). Men experienced an increased risk of advanced stage (Men: 67%; Women: 60%; aRD: 6.7%, 95% CI: -1.7%, 15.1%) for all cancers combined, but this disparity was only statistically significant among patients with anogenital cancers (Men: 72%; Women: 50%; aRD: 22.0%, 95% CI: 0.5%, 43.5%).

**Conclusions:**

Accounting for the types of cancers experienced by men and women strongly attenuated disparities in time to treatment initiation and stage. Higher incidence of rarer cancers among men could explain these disparities.

## Introduction

Global cancer incidence is projected to increase from 17 to 22 million per year from 2015 to 2030, with most new cases and deaths expected to occur in low- and middle- income countries (LMIC) [1,2]. International Agency for Research in Cancer statistics from 2012 revealed that LMICs already experienced the majority of new cancer cases (57%) and deaths (65%) worldwide [3]. Health systems in LMICs are ill-equipped to face this burden due to limited numbers of oncology-trained staff, infrastructure for radiation and surgery, and affordable chemotherapy [4, 5]. In light of these resource constraints, efficient targeting of services is essential to ensure equitable delivery of cancer care [6].

Botswana, a middle-income country in southern Africa, has recorded impressive declines in AIDS-related mortality due to its commitment to universal access to health care and public-private partnerships to scale antiretroviral therapy (ART) programs [7, 8]. Building on these successes, Botswana’s health system is adapting to manage non-communicable diseases, including cancer. In this context of expanded access to ART and an aging population, overall cancer incidence increased, driven primarily by increases in cervical cancer, anogenital cancers, and head and neck cancers [9]. Studies in public and private hospitals in Botswana have found that men in Botswana present with more advanced cancer than women [10–12]. Although reports from other sub-Saharan African countries have assessed risk factors for female cancers (primarily of the breast), less is known about factors associated with delays in cancer care among men [13–18].

We sought to understand whether male oncology patients in Botswana experienced longer time from presentation with cancer symptoms to treatment initiation compared to women, and determine whether those differences could be explained by individual patient factors or cancer type. We further sought to understand whether patient factors or cancer type could explain the disparity in stage at presentation to oncology care between men and women.

## Methods

### Study setting, design, and population

Patients with suspected cancer in Botswana are referred from primary care clinics and district hospitals to two main public tertiary referral centers, Princess Marina Hospital (PMH) in Gaborone and Nyangabgwe Referral Hospital (NRH) in Francistown for specialized oncology care. Specialized oncology care—including chemotherapy, surgery, and radiation—is available to citizens free of cost. Oncology care is also available at two private hospitals, Gaborone Private Hospital (GPH, which serves as the sole radiotherapy center in the country) and Bokamoso Private Hospital (BPH), both located in Gaborone city.

For this analysis, we used data from the Thabatse Cancer Cohort (TCC), which recruits patients from PMH, NRH, GPH and BPH, a sample of patients seeking oncology care in the public and private sector. This cohort, representing an estimated 65% of all cancer cases in the country, was established in 2010 to study cancer survival and progression among patients in Botswana. TCC participants are recruited at their first visit for oncology care and are followed for five years to monitor treatment outcomes. Participants older than 18 years, who are not incarcerated, and agree to participate are eligible for the study. At baseline, TCC study participants are interviewed to retrospectively obtain sociodemographic data, cancer risk factors, and personal history of symptoms and cancer evaluations (S1 File). This study was reviewed and approved by the institutional review board at the Harvard T. H. Chan School of Public Health and the Botswana Ministry of Health. Written documentation of informed consent was obtained from all individual participants included in the study.

We included TCC participants enrolled between October 2010 and June 2017 in this analysis. We excluded cases of Kaposi’s sarcoma due to its unique staging system, and because many Kaposi’s sarcoma patients are treated locally at district hospitals and therefore not referred for specialized oncology care. We restricted the study population to participants who initiated treatment within three years of presenting with their cancer symptom, corresponding to the 90^th^ percentile of observations to limit influence of outliers. We chose to censor at three years rather than using more conventional follow-up time intervals to allow our models to accurately reflect the right skew towards longer follow-up times in this study population. Those with incomplete endpoint and covariate information were also excluded. Excluded participants did not differ from included participants with respect to sex or other covariates.

### Ethical statement

All procedures performed in studies involving human participants were in accordance with the ethical standards of the Harvard T. H. Chan School of Public Health, Botswana Ministry of Health, and with the 1964 Helsinki declaration and its later amendments or comparable ethical standards.

### Outcome, exposure, and covariates

We sought to understand whether the time from first visit for cancer symptom at primary health facility to treatment initiation was different between men and women. This time period combines the “diagnostic” and “pre-treatment” appraisal intervals referred to in the Aarhus statement to standardize reporting across studies on early cancer diagnosis [19]. We refer to this interval, also referred to as the “health system” interval by Unger-Saldaña et al., hereafter as the time-to-treatment [20]. We calculated time-to-treatment as the difference from date of cancer treatment initiation and date of first visit for cancer symptom reported by the participant at time of TCC enrollment, in months. Dates of diagnosis and treatment were recorded from patient medical records. Dates of first visit for cancer symptom were verified in clinical records for 60% of participants, and the remainder were verified using scanned medical cards and pathology records. The date of treatment initiation was the reported date of initiation of chemotherapy, radiotherapy or surgery, whichever came first. For the 231 (n = 11%) participants for whom specific treatment initiation date was missing, we used date of TCC enrollment, which marks initiation of oncology care. Sensitivity analysis incorporating lags from 1 to 3 months to account for additional time between consent and treatment initiation did not change estimates for mean and median time from first clinic visit to treatment initiation. Therefore, we felt that use of consent date could serve as a reasonable estimate for treatment initiation date when missing.

We further studied the difference in proportion of men and women who presented with advanced stage cancers. In Botswana, cancers are diagnosed based on histology of primary tumors (90%), with the remainder made through other histology, cytology/hematology, and clinical examination based on guidelines in similar settings [21]. Specific diagnoses were recorded using ICD-10 codes by clinical staff, and research assistants extracted this information from patient medical records. Stage at diagnosis was recorded in patient medical records based on American Joint Committee on Cancer (AJCC) Guidelines 7 [22]. Cancers with stage of III or IV were considered advanced. A missing indicator variable was created to denote missing stage. With limited therapeutic options, patients with clinically-advanced esophageal cancer seldom completed AJCC staging evaluation, but were considered to have advanced cancer in analysis.

Self-reported biological sex was provided in the baseline TCC questionnaire (male or female). Rather than asking whether there is a true biological effect of sex on time-to-treatment and stage, which requires strong untestable assumptions [23], we instead ask if there is a sex disparity in the length of time-to-treatment, and advanced stage at diagnosis, holding constant factors that are associated with biological sex and our two outcomes.

Covariate data were collected retrospectively through baseline TCC questionnaires. We controlled for patient factors, including sociodemographic and clinical characteristics of patients, which have been associated with health system delays and outcomes in other African settings. [10–14, 24–29].

Sociodemographic characteristics included patient age, poverty (defined as <500 pula/month), educational attainment (primary school or less), marital status (married/cohabiting or other), residence type (city/town or farm/village), household with an indoor toilet, electricity at the home, and use of traditional healer. We also included clinical characteristics including HIV status (considered unknown if no history positive test and no negative test within 2 years), presence of pain symptom at diagnosis, current smoking status, family history of cancer, and cancer type.

To assess self-reported health status, we relied on two questions: “Overall, how would you rate your health during the past 4 weeks?” (binary: Fair, Poor, Very Poor v. Good, Very Good, Excellent), and “During the past 4 weeks, how much did your physical health or emotional problems limit your usual social activities with family or friends?” (binary: No social activities & Quite a lot vs. Somewhat, Very little, Not at all).

We classified cancer type into the following categories: breast, cervical, non-Hodgkin’s lymphoma, esophageal, anogenital (anal, penile, vulvar, vaginal), and other (cancer types with fewer than 45 cases total, including Hodgkin’s lymphoma, colon, prostate, lung, and skin).

### Statistical analysis

We assessed the bivariate association between individual covariates, outcomes, and male sex using chi-square, Student’s t, and Wilcoxon rank sum tests as appropriate. Our primary analytic approach was standardization, which answers the question: “What would be the difference in outcome if everyone in the population had been men, compared to if everyone in the population had been women?” Upon adjustment for sociodemographic factors and clinical characteristics, this approach allows us to quantify sex disparities in length of time-to-treatment and stage by taking the difference in time-to-treatment or risk had everyone been male compared to everyone being female.

To illustrate univariate differences in the length of time-to-treatment and account for non-normally distributed times, we compared median time-to-treatment by sex, stratified by cancer type. Models for log mean length of time-to-treatment in months among men and women were estimated using adjusted accelerated failure time models with a Weibull distribution. For each cancer type, we standardized the sex-specific log mean length of time-to-treatment to the distribution of the following covariates in the patients with that cancer type: age (quadratic), poverty, education, income, marital status, urban/rural residence, presence of indoor toilet at home, presence of electricity at home, use of traditional healer, HIV status, low self-reported health, pain symptom, affirm that disease limits social activities, family history of cancer, ever smoked, and cancer type (for all cancers).

Mean models for proportion of cases with advanced stage were fit using adjusted logistic regression. Predicted probabilities of advanced stage estimated from the logistic regression models were used to estimate risk. For each cancer grouping, we estimated the sex-specific probabilities of advanced stage in men and women, standardized to the distribution of the following covariates in the patients with that cancer type: age (quadratic), poverty, education, income, marital status, urban/rural residence, presence of indoor toilet at home, presence of electricity at home, use of traditional healer, HIV status, family history of cancer, and ever smoked. We only adjusted for cancer type for patients with all cancers. We did not adjust for HIV status for anogenital cancers because HIV status was highly collinear with anogenital cancer status. For the models for advanced stage, we excluded the following variables included in the model for time-to-treatment because they were mediators on the causal path from sex to advanced stage: 1) low self-reported health, 2) pain symptom, and 3) whether disease limited social activities. We reported mean time-to-treatment differences and risk differences on the absolute scale comparing men to women, with 95% confidence intervals obtained through bootstrapping with 1000 samples. We calculated p-values using simple Z-score transformation [30].

All analyses were conducted with SAS 9.4 software (SAS Institute, Cary, NC). All tests were two-tailed, and p < .05 was considered statistically significant. We checked for collinearity between socioeconomic status variables (household items and wealth) and self-reported health variables (low self-reported health, pain symptom, whether illness limited social activities) using chi-square tests and Spearman correlation coefficients.

#### Sensitivity analysis

Since stage was missing for a fifth of study participants, and missing stage was associated with male sex and several other variables, we conducted a sensitivity analysis using inverse probability weighting to adjust for possible selection bias arising from excluding participants with missing data [31]. Briefly, this approach removes non-causal associations between exposure and missingness through the use of weighted regression. Weights are calculated by estimating the inverse of the probability of missing stage, conditional on exposure and covariates using multiple logistic regression. We used the same covariates described for the primary analysis to estimate our weights. To account for correlated data induced by weighting, we report these results using robust standard errors (S1 Table).

## Results

After excluding cohort participants with missing covariate data (n = 73, 3%) and those whose time to treatment initiation was over the 90^th^ percentile (n = 215, 10%), 1886/2174 (87%) cohort participants contributing 17,194.4 person-months of follow-up remained in our study, of whom 28% were male (Table 1). Participants were followed for a median of 6.4 months [IQR: 3.5–12.5]. Men were older, had lower educational attainment, and were more likely to be in a cohabiting couple than women. Men were more likely to report use of a traditional healer prior to diagnosis, severe cancer-related symptoms, and history of smoking. Men were also less likely to be HIV-infected compared to women in the study population.

**Table 1:** Descriptive characteristics of study population.

	Male (N = 531)		Female (N = 1355)		All (N = 1886)	
	N	%	N	%	N	%
**Age (median, IQR)**	55.76	[43.99–64.6]	47.92	[39.32–60.18]	49.78	[40.24–61.54]
**Poverty (<500 P/mo)**	289	54.43	785	57.93	1074	56.95
**Primary school or less**	333	62.71	703	51.88	1036	54.93
**Cohabiting couple (married/co)**	288	54.24	468	34.54	756	40.08
**Reside in city/town**	420	79.1	1095	80.81	1515	80.33
**Interior toilet at home**	183	34.46	527	38.89	710	37.65
**Electricity at home**	355	66.85	933	68.86	1288	68.29
**Used Traditional healer**	214	40.3	351	25.9	565	29.96
**HIV Status**						
Negative	287	54.05	607	44.8	894	47.4
Positive	216	40.68	687	50.7	903	47.88
Unknown	28	5.27	61	4.5	89	4.72
**Low reported health**	397	74.76	760	56.09	1157	61.35
**Presence of pain symptom**	169	31.83	232	17.12	401	21.26
**Illness limits social activities**	144	27.12	321	23.69	465	24.66
**Family history of cancer**	89	16.76	283	20.89	372	19.72
**Ever smoked**	326	61.39	108	7.97	434	23.01
**Cancer Type**						
Breast	7	1.32	383	28.27	390	20.68
Cervix	-	-	547	40.37	547	29
NHL	62	11.68	47	3.47	109	5.78
Anogenital	69	12.99	89	6.57	158	8.38
Head and Neck	125	23.54	17	1.25	142	7.53
Esophageal	42	7.91	32	2.36	74	3.92
Other	226	42.56	240	17.71	466	24.71

The distribution of cancers varied by sex. Breast (40%) and cervix (28%) were the most common cancer sites for women. Men were most likely to be diagnosed with other less common cancers (43%), head and neck (24%), anogenital (13%) and NHL (12%).

**Fig 1:**
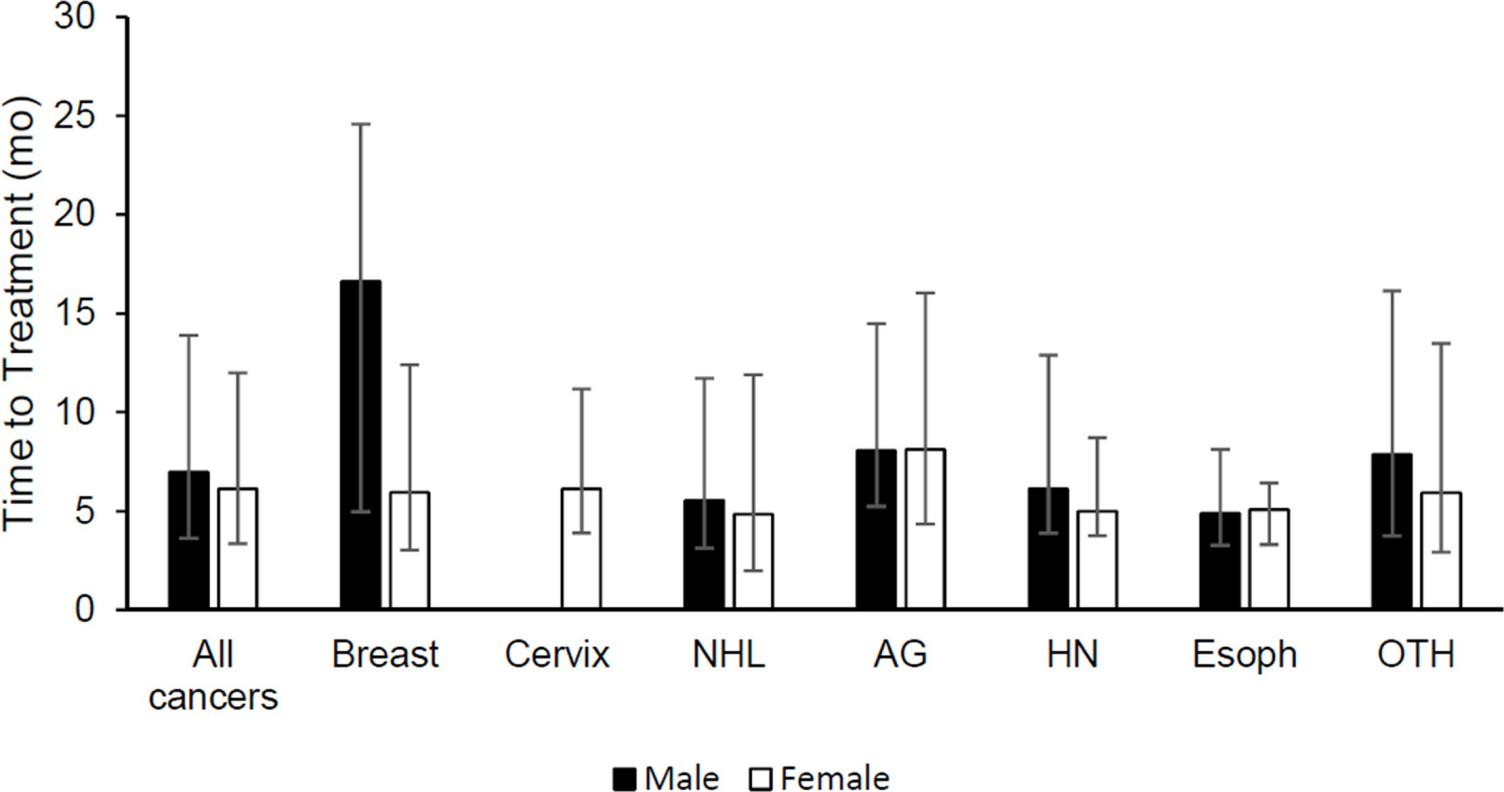
Median time from first presentation with cancer symptom to entry into oncology care in men and women stratified by cancer type. Error bars reflect interquartile ranges. NHL = non-Hodgkin’s lymphoma, AG = anogenital, HN = head and neck, Esoph = Esophageal, OTH = other cancers.

In unadjusted analysis, we found that men experienced significantly longer median time-to-treatment compared to women (men: 7.0 months, [IQR: 3.6–13.9], women: 6.1 months, [IQR: 3.4–12.0], log-rank p = 0.0184). We also found significant differences in median time-to-treatment by cancer type (Fig 1, log-rank p<0.0001). Anogenital cancers took a median of 8.1 months [IQR: 4.3–16.0] for women and 8.1 months [IQR: 5.2–14.5] for men. Initiation of treatment for NHL took a median of 4.8 months [IQR: 2.0–11.9] for women and 5.6 months [IQR: 3.1–11.7] for men. We found a significant difference in median time-to-treatment between men (7.9 months, [IQR: 3.7–16.1] and women (5.9 months, [IQR: 2.9–13.5]) for other non-common cancers (log-rank p = 0.0492).

**Fig 2:**
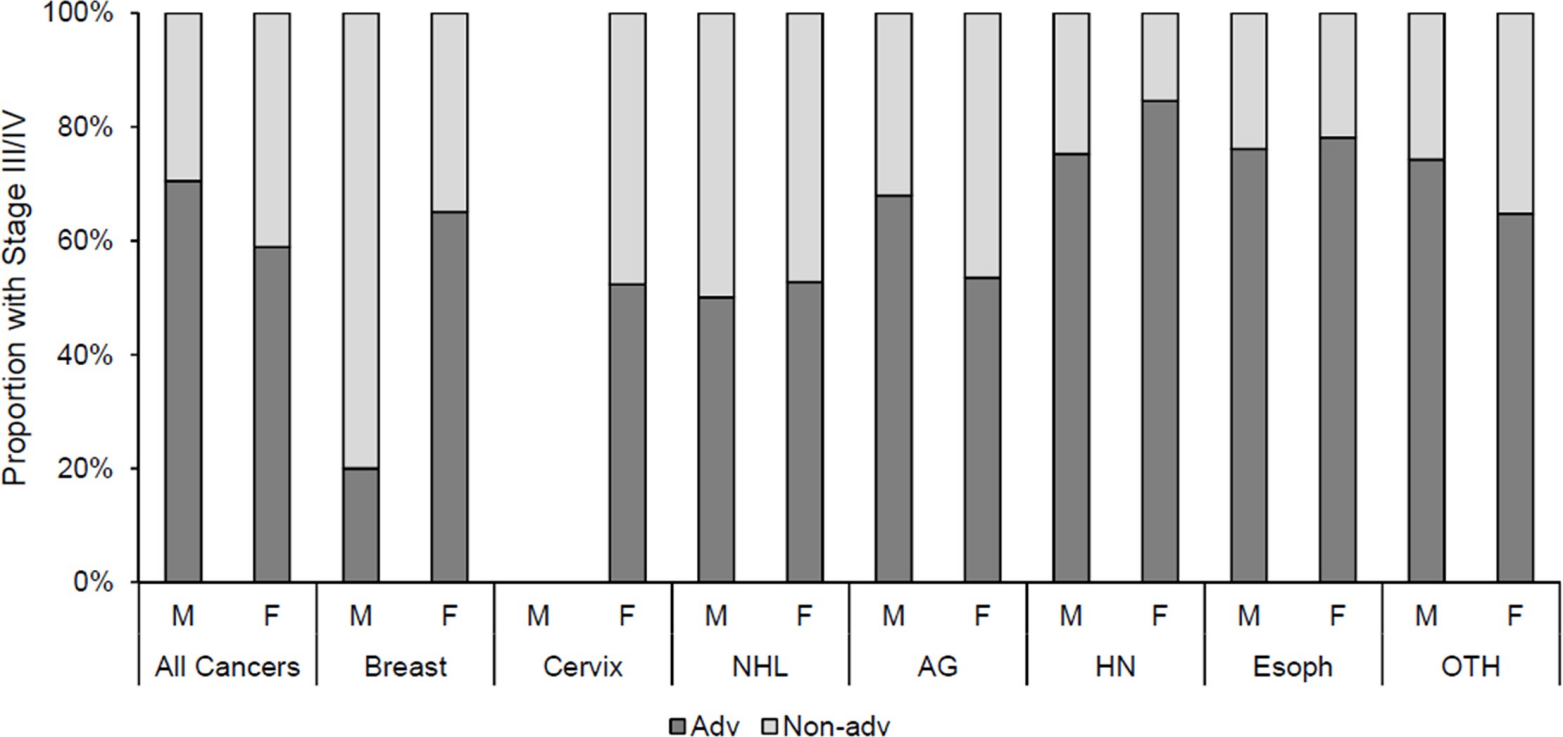
Proportion of advanced stage cancer in men and women stratified by cancer type. NHL = non-Hodgkin’s lymphoma, AG = anogenital, HN = head and neck, Esoph = Esophageal, OTH = other cancers.

Similarly, we found that men experienced a higher proportion of advanced stage cases compared to women (men: 70.5%, women: 58.9%, p<0.0001), and proportion of advanced stage varied by by cancer type (p<0.0001). We studied whether the associations between sex and advanced cancer varied within a given type of cancer (Fig 2) and detected no significant associations. Among staged cancers, we found the highest proportion of advanced stage among those with head and neck (women: 85%, men: 75%) and esophageal cancer (women: 78%, men: 76%). Patients with NHL had the lowest proportion of advanced stage (women: 53%, men: 50%).

**Fig 3:**
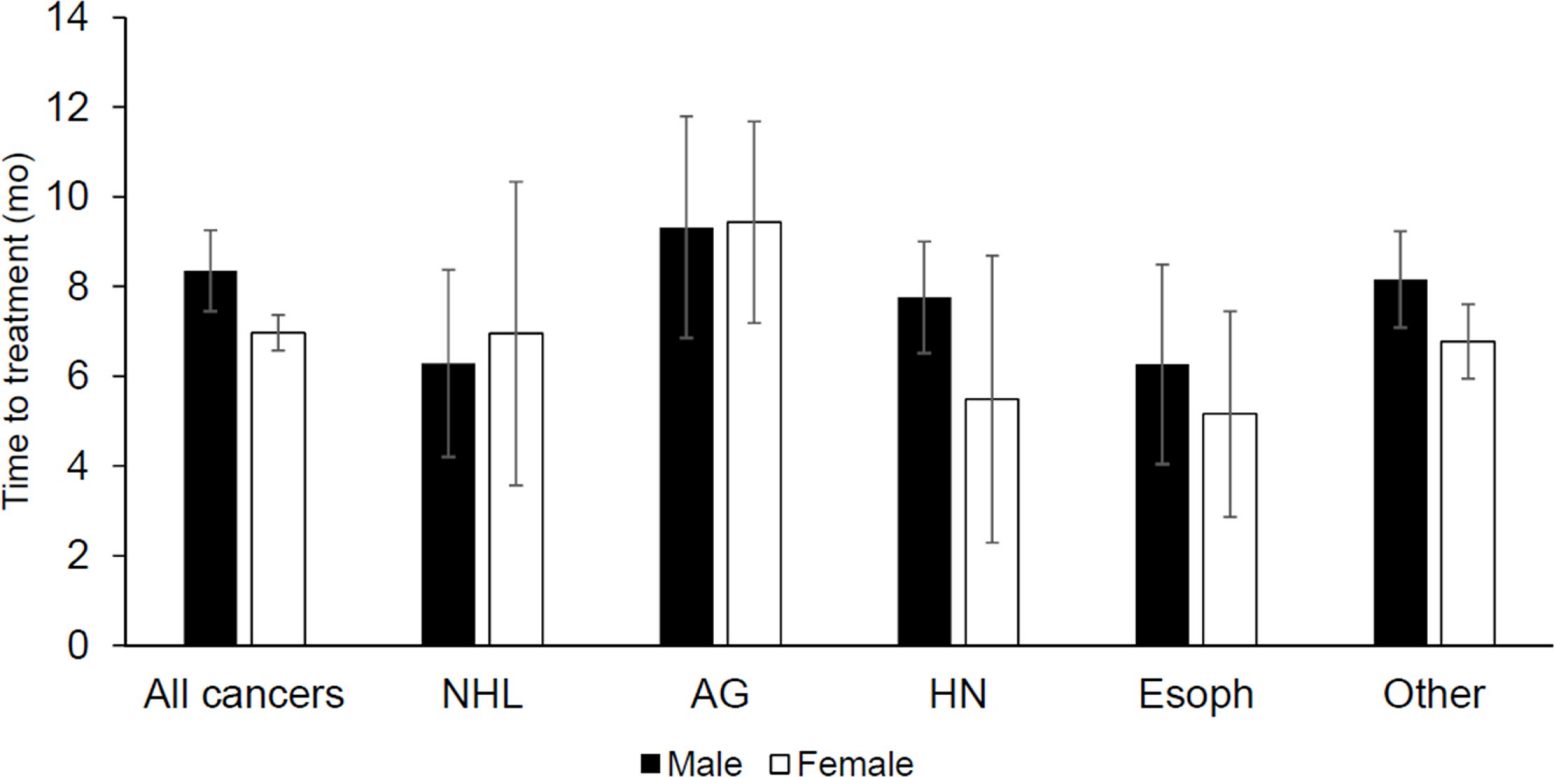
Standardized mean time-to-treatment interval times estimated from accelerated failure time models with Weibull distribution. NHL = non-Hodgkin’s lymphoma, AG = anogenital, HN = head and neck, Esoph = Esophageal, OTH = other cancers. Each cancer grouping was standardized to the distribution of the following covariates in the patients with that cancer type: age (quadratic), poverty, education, income, marital status, urban/rural residence, presence of indoor toilet at home, presence of electricity at home, use of traditional healer, HIV status, low self-reported health, pain symptom, affirm that disease limits social activities, family history of cancer, ever smoked, and cancer type (for the model with all cancers only). All cancers include every cancer type. Other cancer type excludes breast and cervical cancer in addition to the others listed. Error bars reflect 95% confidence intervals for mean time-to-treatment estimates.

In adjusted analysis, we estimated the standardized difference in mean time-to-treatment if all patients had the same distribution of covariates as men compared to if all patients had the same distribution of covariates as women for each of the cancer types common to both sexes (Fig 3, S1 Table). We found that after adjusting for confounding variables, men experienced a significant 1.4 month (95% CI: 0.30, 2.5, p = 0.013) longer time-to-treatment compared to women. When we looked within cancer types, men experienced a 0.11 month time-to-treatment than women among patients with anogenital cancers (95% CI: -3.8, 3.5, p = 0.96), and a 0.66 month (95% CI: -5.2, 3.9, p = 0.79) shorter time-to-treatment among patients with NHL. Men experienced a 2.3 month (95% CI: -1.4, 6.0, p = 0.23) longer time-to-treatment than women among patients with head and neck cancer, and a 1.1 month (-2.8, 5.0, p = 0.59) longer time-to-treatment among patients with esophageal cancer. Among patients with other non-common cancers, men experienced a significant 1.39 month (95% CI: 0.01, 2.76, p = 0.047) longer time-to-treatment than women.

**Fig 4:**
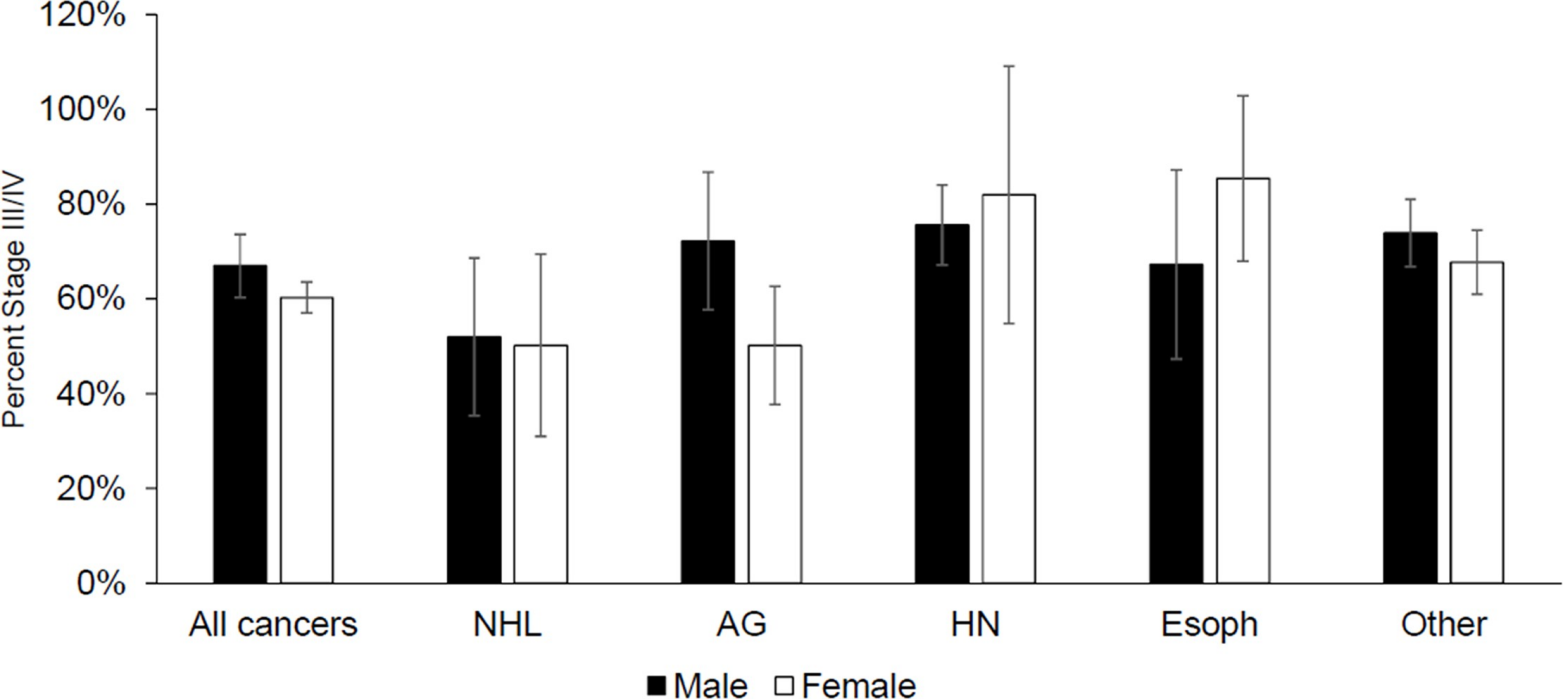
Standardized probabilities of advanced (III/IV) stage estimated from logistic regression models. NHL = non-Hodgkin’s lymphoma, AG = anogenital, HN = head and neck, Esoph = Esophageal, OTH = other cancers. Each cancer grouping was standardized to the distribution of the following covariates in the patients with that cancer type: age (quadratic), poverty, education, income, marital status, urban/rural residence, presence of indoor toilet at home, presence of electricity at home, use of traditional healer, HIV status, family history of cancer, and ever smoked. We only adjusted for cancer type for patients with all cancers. We did not adjust for HIV status for anogenital cancers because HIV status was highly collinear with anogenital cancer status. All cancers include every cancer type. Other cancer type excludes breast and cervical cancer in addition to the others listed. Error bars reflect 95% confidence intervals for the proportion of cases with advanced stage. Some of the 95% confidence intervals include values over 100% due to large standard errors.

After using standardization to adjust for confounding, we further assessed the association between sex and advanced stage, stratified by cancer type (Fig 4, S2 Table). Sensitivity analyses using inverse probability of censoring weights to adjust for possible selection bias did not appreciably change effect estimates so we present results from the complete case analysis (S3 Table). Among patients with all cancers combined, we found a modest, non-significant increased risk of advanced stage cancer among men (aRD: 6.7%, 95% CI: -1.7%, 15.1%, p = 0.12). Though we observed modest within-cancer differences in advanced stage by sex, we only found a significant increased risk of advanced stage cancer among men for patients with anogenital cancers (aRD: 22.0%, 95% CI: 0.5%, 43.5%, p = 0.045). Among patients with esophageal cancer, men experienced an 18.2% (95% CI: -50.6%, 14.3%, p = 0.28) reduced risk of advanced stage compared to women, but this was not statistically significant.

## Discussion

Building on accumulating evidence from Botswana suggesting that men experience longer diagnostic and time-to-treatment and are diagnosed at more advanced stage than women, we were able to exploit a larger cohort study with detailed information on cancer types and sociodemographic factors to explore specific drivers of these disparities. On average, men were more likely than women to present with anogenital and other less common cancers, diseases which exhibited longer diagnostic and treatment appraisal times, as well as more advanced stage at presentation. When we estimated covariate-standardized associations between sex, appraisals, and advanced cancer stratified by cancer type, we found that disparities were strongly attenuated within most cancer types. Significant male-female disparities in time-to-treatment remained only among those patients with uncommon cancers (which tended to be specific to either men or women), and remained among patients with anogenital cancers with respect to stage.

Sex differences in stage could be explained by oncology care seeking patterns that differ between men and women [32]. Women in Botswana engage with the health system more frequently than men for care related to pregnancy and dependents. In addition, men might recognize emergent cancer symptoms more slowly, or take longer to seek care than women. HIV researchers have observed that, in sub-Saharan African contexts, men are less likely than women to engage with HIV testing and remain in ART programs, potentially due to stigma and other economic barriers to care [33]. If sex-specific care-seeking norms extend from HIV to cancer, these same factors could explain the more advanced stage at presentation of men compared to women. Research on sex-specific survival following ART initiation across sub-Saharan Africa consistently demonstrates poorer survival in men than women. These disparities are not fully explained by differences in demographic and clinical characteristics [34–36]. Researchers suggest that these differences could be due to biological differences of ART effectiveness, or poorer adherence to treatment among male patients compared to female patients. Others have argued that by emphasizing maternal and child interventions, HIV control programs have inadvertently neglected opportunities to engage men in care [37]. In our study, we similarly found that control for sociodemographic and clinical characteristics did not fully explain away the sex disparity in time-to-treatment, but once different cancer types were accounted for, most of the disparity was eliminated. This finding supports the notion that differences in prognosis for men compared to women could be driven by patterns of cancer each group experiences rather than biological differences.

These findings have several implications for population-level interventions to control cancer in Botswana. First, men should be encouraged to engage with the health system more quickly to improve prognosis. Community sensitization campaigns targeted at men could help men with suspected cancer symptoms present earlier to care. Second, efforts should be made to equip health centers, hospitals, and providers with the training and technology they need to diagnose less common cancers more quickly. Botswana has established a national cervical cancer control strategy through implementation of visual inspection acetic acid screening and treatment program [17, 38, 39]. Non-governmental organizations like Journey of Hope, along with individual health providers are similarly developing programs to reduce breast cancer mortality [40]. Given that men with HIV have regular health system contact and that HIV remains a major risk factor for cancer in Botswana [9], routine HIV care also represents an opportunity to facilitate prompt evaluation of cancer-related signs and symptoms.

Our study had a number of limitations. We have previously found that men in Botswana are substantially less likely to receive treatment for their cancer than women. The current analysis is limited to those who successfully entered oncology care and may not reflect the experiences and disparities encountered for those men with cancer who are unable to access care. Date of initial clinic presentation was verified by clinical records when present, but the measurement of time-to-treatment remains vulnerable to recall bias. Confounding by unmeasured factors could remain, including those related to the health system, patient healthcare utilization, and patient beliefs regarding cancer, though adjustment for sociodemographic and clinical characteristics associated with these factors would reduce bias. Strengths of this study include the size of this unique study population, allowing us to study sex disparities within cancer types, detailed information on timing of visits along the cancer care continuum, and covariate information collected for study participants related to their care, demographics, socioeconomic status, and clinical factors. While Botswana’s cancer burden reflects its unique history in combatting HIV, leading to specific patterns of cancer incidence, other sub-Saharan African countries may also find oncology care disparities related to sex that could be explained by a higher prevalence of cancers in men which are not explicitly targeted by the health system.

In summary, we find that differences in health system delay and advanced stage among male compared to female cancer patients in Botswana appear to be mainly driven by differences in the types of cancers that arise within each sex. Interventions that raise awareness of cancer symptoms among men and sensitization of health providers to cancers common in men could help reduce the length of time between first clinic visit with oncology related system and treatment initiation for all cancer patients, and hopefully improve prognosis for male patients in Botswana. Future studies should explore the role of patient beliefs and health system factors on delays and stage at presentation.

## Supporting information

S1 Table Standardized mean time-to-treatment interval times (months) and 95% confidence intervals for men and women estimated from accelerated failure time models with Weibull distribution. (DOCX)

S2 Table Standardized probabilities and 95% confidence intervals of advanced (III/IV) stage for men and women estimated from logistic regression models*. *Note some of the confidence interval upper bounds are greater than 1 due to bootstrapped sampling.
(DOCX)

S3 Table Standardized risk difference estimates of advanced (stage III/IV) cancer among men and women in Botswana. (DOCX)

S1 File Thabatse cancer cohort survey questionnaire. (PDF)
