# Supplementary material for: Explaining disparities in oncology health systems delays and stage at diagnosis between men and women in Botswana: A cohort study
Source: PLoS One. 2019 Jun 6;14(6):e0218094. doi: 10.1371/journal.pone.0218094 (PMC6553768; doi:10.1371/journal.pone.0218094)
Supplement: S1 Table — (DOCX) [file pone.0218094.s001.docx]

**Supplementary Table 1. Standardized Risk Difference Estimates of Advanced (Stage III/IV) Cancer among Men and Women in Botswana**

|  | **No censoring weights** | | **Censoring weights** | |
| --- | --- | --- | --- | --- |
| **Cancer type** | **Risk Difference** | **95% CI** | **Risk Difference** | **95% CI** |
| All cancers | 0.067 | -0.017, 0.15 | 0.071 | -0.014, 0.16 |
| Anogenital | 0.22 | 0.005, 0.44 | 0.23 | 0.011, 0.44 |
| NHL | 0.019 | -0.27, 0.31 | 0.032 | -0.26, 0.33 |
| HN | -0.063 | -0.35, 0.23 | -0.011 | -0.33, 0.31 |
| Esophagus | -0.18 | -0.51, 0.14 | - | - |
| Other (excl breast and cervix) | 0.062 | -0.047, 0.17 | 0.049 | -0.060, 0.16 |
