# Supplementary material for: Explaining disparities in oncology health systems delays and stage at diagnosis between men and women in Botswana: A cohort study
Source: PLoS One. 2019 Jun 6;14(6):e0218094. doi: 10.1371/journal.pone.0218094 (PMC6553768; doi:10.1371/journal.pone.0218094)
Supplement: S2 Table — *Note some of the confidence interval upper bounds are greater than 1 due to bootstrapped sampling. (DOCX) [file pone.0218094.s002.docx]

**Supplementary Table 2. Standardized Mean Time-to-Treatment Interval Times (Months) and 95% confidence intervals for men and women estimated from accelerated failure time models with Weibull distribution**

| **Cancer type** | **Men** | **Women** |
| --- | --- | --- |
| **All cancers** | 8.35 (7.45, 9.25) | 6.97 (6.58, 7.37) |
| **Non-Hodgkin’s Lymphoma** | 6.29 (4.21, 8.37) | 6.95 (3.57, 10.34) |
| **Anogenital** | 9.32 (6.86, 11.79) | 9.44 (7.19, 11.68) |
| **Head and Neck** | 7.76 (6.51, 9.01) | 5.49 (2.29, 8.69) |
| **Esophageal** | 6.27 (4.04, 8.49) | 5.16 (2.87, 7.45) |
| **Other** | 8.16 (7.08, 9.23) | 6.77 (5.94, 7.61) |
