# Supplementary material for: Explaining disparities in oncology health systems delays and stage at diagnosis between men and women in Botswana: A cohort study
Source: PLoS One. 2019 Jun 6;14(6):e0218094. doi: 10.1371/journal.pone.0218094 (PMC6553768; doi:10.1371/journal.pone.0218094)
Supplement: S3 Table — (DOCX) [file pone.0218094.s003.docx]

**Supplementary Table 3. Standardized Probabilities and 95% confidence intervals of Advanced (III/IV) Stage for men and women estimated from logistic regression models***

| **Cancer type** | **Men** | **Women** |
| --- | --- | --- |
| **All cancers** | 0.67 (0.60, 0.74) | 0.60 (0.57, 0.64) |
| **Non-Hodgkin’s Lymphoma** | 0.52 (0.35, 0.69) | 0.50 (0.31, 0.69) |
| **Anogenital** | 0.72 (0.58, 0.87) | 0.50 (0.38, 0.63) |
| **Head and Neck** | 0.76 (0.67, 0.84) | 0.82 (0.55, 1.09) |
| **Esophageal** | 0.67 (0.47, 0.87) | 0.85 (0.68, 1.03) |
| **Other** | 0.74 (0.67, 0.81) | 0.68 (0.61, 0.74) |

***Note some of the confidence interval upper bounds are greater than 1 due to bootstrapped sampling**
