# Supplementary material for: Explaining disparities in oncology health systems delays and stage at diagnosis between men and women in Botswana: A cohort study
Source: PLoS One. 2019 Jun 6;14(6):e0218094. doi: 10.1371/journal.pone.0218094 (PMC6553768; doi:10.1371/journal.pone.0218094)
Supplement: S1 File — (PDF) [file pone.0218094.s004.pdf]

BHP045 Edc Exported Form Text: Cancer Subject Package 20160308

Forms for cancer_subject

Subject Visit

Edc Docstring: SubjectVisit(created, modified, user_created, user_modified, hostname_created, hostname_modified, id, revision, appointment_id, report_datetime, reason, reason_missed, info_source, info_source_other, comments, subject_identifier, reason_unscheduled)

Instructions: Please complete the questions below.

1. appointment

cancer_subject_subjectvisit.appointment

- dropdown [Appointment]

2. Visit Date and Time

cancer_subject_subjectvisit.report_datetime

____

Date and time of this report

3. What is the main source of this information?

cancer_subject_subjectvisit.info_source

____

4. ...if "Other", specify

cancer_subject_subjectvisit.info_source_other

____

5. What is the reason for this visit?

cancer_subject_subjectvisit.reason

____

<Override the field class for this model field attribute in ModelForm>

6. If 'Unscheduled' above, provide reason for the unscheduled visit

cancer_subject_subjectvisit.reason_unscheduled

- Routine oncology clinic visit (i.e. planned chemo, follow-up)
- Ill oncology clinic visit
- Patient called to come for visit
- Other, specify:

7. If 'missed' above, Reason scheduled visit was missed

cancer_subject_subjectvisit.reason_missed

____

8. Comment if any additional pertinent information about the participant

cancer_subject_subjectvisit.comments

____

Exported from Edc. Revision tag: 1.1.15

Subject Consent

Edc Docstring: SubjectConsent(created, modified, user_created, user_modified, hostname_created, hostname_modified, id, revision, subject_identifier_as_pk, subject_identifier_aka, dm_comment, first_name, last_name, initials, dob, is_dob_estimated, gender, subject_type, subject_identifier, study_site_id, consent_datetime, guardian_name, may_store_samples, is_incarcerated, is_literate, witness_name, comment, consent_version_on_entry, consent_version_recent, language, is_verified, is_verified_datetime, consent_reviewed, study_questions, assessment_score, consent_copy, identity, identity_type, confirm_identity, registered_subject_id, original_identifier)

Instructions: Please complete the questions below.

1. Subject Identifier

cancer_subject_subjectconsent.subject_identifier

____

2. first name

cancer_subject_subjectconsent.first_name

____

(Encryption: rsa local)

3. Last name

cancer_subject_subjectconsent.last_name

____

(Encryption: rsa restricted)

4. initials

cancer_subject_subjectconsent.initials

____

(Encryption: rsa local)

5. Consent date and time

cancer_subject_subjectconsent.consent_datetime

____

6. Gender

cancer_subject_subjectconsent.gender

- Male
- Female
- Undetermined

7. Site

cancer_subject_subjectconsent.study_site

- dropdown [StudySite]

This refers to the site or 'clinic area' where the subject is being consented.

8. Is the participant LITERATE?

cancer_subject_subjectconsent.is_literate

- Yes
- No

(if 'No' provide witness's name here and with signature on the paper document.)

9. Witness's Last and first name (illiterates only)

cancer_subject_subjectconsent.witness_name

____

Required only if subject is illiterate. Format is 'LASTNAME, FIRSTNAME'. All uppercase separated by a comma (Encryption: rsa restricted)

10. Date of birth

cancer_subject_subjectconsent.dob

____

Format is YYYY-MM-DD

11. Is date of birth estimated?

cancer_subject_subjectconsent.is_dob_estimated

- No
- Yes, estimated the Day
- Yes, estimated Month and Day
- Yes, estimated Year, Month and Day

If the exact date is not known, please indicate which part of the date is estimated.

12. Identity number (OMANG, etc)

cancer_subject_subjectconsent.identity

____

Use Omang, Passport number, driver's license number or Omang receipt number (Encryption: rsa restricted)

13. What type of identity number is this?

cancer_subject_subjectconsent.identity_type

- Omang
- Driver's License
- Passport
- Omang Receipt
- Other

14. confirm identity

cancer_subject_subjectconsent.confirm_identity

____

Retype the identity number from the identity card (Encryption: rsa restricted)

15. Is the participant under involuntary incarceration?

cancer_subject_subjectconsent.is_incarcerated

- Yes
- No

(if 'YES' STOP patient cannot be consented)

16. Comment

cancer_subject_subjectconsent.comment

____

(Encryption: aes local)

17. I have reviewed the consent with the client

cancer_subject_subjectconsent.consent_reviewed

- Yes
- No

If no, INELIGIBLE

18. I have answered all questions the client had about the study

    cancer_subject_subjectconsent.study_questions

- Yes
- No

If no, INELIGIBLE

19. I have asked the client questions about this study and they have demonstrated understanding

    cancer_subject_subjectconsent.assessment_score

- Yes
- No

If no, INELIGIBLE

20. I have provided the client with a copy of their signed informed consent

    cancer_subject_subjectconsent.consent_copy

- Yes
- No
- Yes, but subject declined copy

If declined, return copy to the clinic with the consent

Exported from Edc. Revision tag: 1.1.15

Enrollment Site

Edc Docstring: EnrollmentSite(id, created, modified, user_created, user_modified, hostname_created, hostname_modified, site_name)

Instructions: Please complete the questions below.

Exported from Edc. Revision tag: 1.1.15

Subject Death

Edc Docstring: SubjectDeath(created, modified, user_created, user_modified, hostname_created, hostname_modified, id, revision, registered_subject_id, death_date, death_cause_info_id, death_cause_info_other, death_cause, death_cause_category_id, death_cause_other, participant_hospitalized, death_reason_hospitalized_id, days_hospitalized, comment, is_death_date_estimated, subject_visit_id)

Instructions: Please complete the questions below.

1. registered subject

    cancer_subject_subjectdeath.registered_subject

- dropdown [RegisteredSubject]

2. subject visit

    cancer_subject_subjectdeath.subject_visit

- dropdown [SubjectVisit]

3. Date of Death:

    cancer_subject_subjectdeath.death_date

____

4. Is date of death estimated?

cancer_subject_subjectdeath.is_death_date_estimated

- No
- Yes, estimated the Day
- Yes, estimated Month and Day
- Yes, estimated Year, Month and Day

If the exact date is not known, please indicate which part of the date is estimated.

5. What is the primary source of cause of death information? (if multiple source of information, list one with the smallest number closest to the top of the list)

cancer_subject_subjectdeath.death_cause_info select multiple options

- No information will ever be available
- Autopsy
- Clinical record
- Information from physician/nurse/other health care provider
- Information from participant's relatives or friends
- Information requested, still pending
- Other, specify

6. if other specify...

cancer_subject_subjectdeath.death_cause_info_other

____

7. Describe the major cause of death(including pertinent autopsy information if available),starting with the first noticeable illness thought to be related to death,continuing to time of death.

cancer_subject_subjectdeath.death_cause

____

Note: Cardiac and pulmonary arrest are not major reasons and should not be used to describe major cause)

8. Based on the above description, what category best defines the major cause of death?

cancer_subject_subjectdeath.death_cause_category select multiple options

- No information will ever be available
- Cancer
- HIV infection or HIV/AIDS-related diagnosis
- Disease/injury unrelated to cancer or HIV
- Toxicity from cancer treatment (complications of chemotherapy, radiation, or surgery)
- Toxicity from HIV/AIDS treatment (HAART or treatment of HIV/AIDS-related diagnosis)
- Other, specify

9. if other specify...

cancer_subject_subjectdeath.death_cause_other

____

10. Comments

cancer_subject_subjectdeath.comment

____

Exported from Edc. Revision tag: 1.1.15

Subject Off Study

Edc Docstring: SubjectOffStudy(created, modified, user_created, user_modified, hostname_created, hostname_modified, id, revision, registered_subject_id, offstudy_date, reason, reason_other, has_scheduled_data, comment, subject_visit_id)

Instructions: Please complete the questions below.

1. registered subject

cancer_subject_subjectoffstudy.registered_subject

- dropdown [RegisteredSubject]

2. subject visit

cancer_subject_subjectoffstudy.subject_visit

- dropdown [SubjectVisit]

3. Off-study Date

cancer_subject_subjectoffstudy.offstudy_date

---

4. Please code the primary reason participant taken off-study

cancer_subject_subjectoffstudy.reason

---

5. ...if "Other", specify

cancer_subject_subjectoffstudy.reason_other

---

6. Are scheduled data being submitted on the off-study date?

cancer_subject_subjectoffstudy.has_scheduled_data

- Yes
- No

7. Comments:

cancer_subject_subjectoffstudy.comment

Exported from Edc. Revision tag: 1.1.15

Enrollment Checklist

Edc Docstring: EnrollmentChecklist(created, modified, user_created, user_modified, hostname_created, hostname_modified, id, revision, registered_subject_id, registration_datetime, has_diagnosis, enrollment_site_id)

Instructions: Please complete the questions below.

1. registered subject

cancer_subject_enrollmentchecklist.registered_subject

- dropdown [RegisteredSubject]

2. Today's date

cancer_subject_enrollmentchecklist.registration_datetime

---

3. Has a cancer diagnosis been documented?

cancer_subject_enrollmentchecklist.has_diagnosis

- Yes
- No

(if 'NO' STOP patient cannot be enrolled)

4. enrollment site

    cancer_subject_enrollmentchecklist.enrollment_site

- dropdown [EnrollmentSite]

Hospital where subject is recruited

Exported from Edc. Revision tag: 1.1.15

Base Risk Assessment

Edc Docstring: CA001

Instructions: Please complete the questions below.

1. subject visit

    cancer_subject_baseriskassessment.subject_visit

- dropdown [SubjectVisit]

2. Have you been told you have hepatitis B or C before?

    cancer_subject_baseriskassessment.hepatitis

- No
- Hepatitis B
- Hepatitis C
- Don't know

3. Do you have now or have you ever had tuberculosis?

    cancer_subject_baseriskassessment.tuberculosis

- Yes
- No
- Do not Know

4. In what year did you last have tuberculosis (year of diagnosis)?

    cancer_subject_baseriskassessment.year_tb

    ____

5. Have you ever worked at a mine?

    cancer_subject_baseriskassessment.has_worked_mine

- Yes
- No
- Patient declined to answer

6. Have you ever smoked cigarettes?

    cancer_subject_baseriskassessment.has_smoked

- Yes
- No
- Patient declined to answer

7. How old were you when you first had sex?

    cancer_subject_baseriskassessment.age_firstsex

- younger than 15 years old
- between 15 and 17 years old
- older than 17 years old
- don't know
- never
- Don't want to answer
- Patient declined to answer

8. Do you drink alcohol?

cancer_subject_baseriskassessment.has_alcohol

- Yes
- No
- Patient declined to answer

9. How often do you use traditional medicine?

cancer_subject_baseriskassessment.tradmedicine

- Never
- Less than once a year
- Between 1 and 5 times a year
- Between 5 to 10 times a year
- More than 10 times a year
- Patient declined to answer

10. Is patient an albino?

cancer_subject_baseriskassessment.is_albino

- Yes
- No

Interform rules (Rule Groups)

1. BaseRiskAssessmentRuleGroup.has_smoked()
   - Returns True if has_smoked equals No OR has_smoked equals Declined. If True sets *Base Risk Assessment: Smoking* to 'not_required' otherwise 'new'.
2. BaseRiskAssessmentRuleGroup.has_alcohol()
   - Returns True if has_alcohol equals No OR has_alcohol equals Declined. If True sets *Base Risk Assessment: Alcohol* to 'not_required' otherwise 'new'.
3. BaseRiskAssessmentRuleGroup.has_worked_mine()
   - Returns True if has_worked_mine equals No OR has_worked_mine equals Declined. If True sets *Base Risk Assessment: Mining* to 'not_required' otherwise 'new'.

Exported from Edc. Revision tag: 1.1.15

Base Risk Assessment: Smoking

Edc Docstring: BaseRiskAssessmentSmoking(created, modified, user_created, user_modified, hostname_created, hostname_modified, id, revision, subject_visit_id, report_datetime, smoke_now, cigarette_smoking, years_smoked, cigarette_smoked, when_quit, years_smoked_before)

Instructions: Please complete the questions below.

1. subject visit

cancer_subject_baseriskassessmentsmoking.subject_visit

- dropdown [SubjectVisit]

2. Do you smoke cigarettes now?

cancer_subject_baseriskassessmentsmoking.smoke_now

- yes
- no, I used to smoke but quit

3. How many cigarettes do you smoke per day?

cancer_subject_baseriskassessmentsmoking.cigarette_smoking

- 14 or fewer cigarettes a day
- between 15 and 25 cigarettes a day
- more than 25 cigarettes a day
- Participant declined to answer

4. For how many years have you smoked?

cancer_subject_baseriskassessmentsmoking.years_smoked

____

5. How many cigarettes did you smoke per day?

cancer_subject_baseriskassessmentsmoking.cigarette_smoked

- 14 or fewer cigarettes a day
- between 15 and 25 cigarettes a day
- more than 25 cigarettes a day
- Participant declined to answer

6. When did you quit smoking cigarettes?

cancer_subject_baseriskassessmentsmoking.when_quit

- less than 2 years ago
- between 2 and 10 years ago
- between 10 and 20 years ago
- more than 20 years ago
- Participant declined to answer

7. For how many years did you smoke before quitting?

cancer_subject_baseriskassessmentsmoking.years_smoked_before

____

Exported from Edc. Revision tag: 1.1.15

Base Risk Assessment: Sun

Edc Docstring: BaseRiskAssessmentSun(created, modified, user_created, user_modified, hostname_created, hostname_modified, id, revision, subject_visit_id, report_datetime, hours_outdoor, sleeved_shirt, hat, shade_umbrella, sunglasses)

Instructions: Please complete the questions below.

1. subject visit

cancer_subject_baseriskassessmentsun.subject_visit

- dropdown [SubjectVisit]

2. On average, how many hours are you outdoors per day between 10am and 4pm?

cancer_subject_baseriskassessmentsun.hours_outdoor

- 1 hour or less
- 2 hours
- 3 hours
- 4 hours
- 5 hours
- 6 hours

3. When you are outside on a sunny day, how often do you wear a SHIRT WITH SLEEVES?

cancer_subject_baseriskassessmentsun.sleeved_shirt

- never
- rarely
- sometimes
- often
- always

4. When you are outside on a sunny day, how often do you wear a HAT?

cancer_subject_baseriskassessmentsun.hat

- never
- rarely
- sometimes
- often
- always

5. When you are outside on a sunny day, how often do you stay in the SHADE or UNDER AN UMBRELLA?

cancer_subject_baseriskassessmentsun.shade_umbrella

- never
- rarely
- sometimes
- often
- always

6. When you are outside on a sunny day, how often do you wear SUNGLASSES?

cancer_subject_baseriskassessmentsun.sunglasses

- never
- rarely
- sometimes
- often

- always

Exported from Edc. Revision tag: 1.1.15

Base Risk Assessment: Mining

Edc Docstring: BaseRiskAssessmentMining(created, modified, user_created, user_modified, hostname_created, hostname_modified, id, revision, subject_visit_id, report_datetime, mine_time, mine_type, mine_prompt_other, mine_underground, mine_underground_time, last_mine)

Instructions: Please complete the questions below.

1. subject visit

cancer_subject_baseriskassessmentmining.subject_visit

- dropdown [SubjectVisit]

2. 43. What is the total amount of time you worked in the mine?

cancer_subject_baseriskassessmentmining.mine_time

- less than 5 years
- between 5 and 20 years
- more than 20 years

3. 44. What kind of mine have you worked in?

cancer_subject_baseriskassessmentmining.mine_type

- gold
- diamond
- copper
- nickel
- other, specify:

4. ...if "Other", specify

cancer_subject_baseriskassessmentmining.mine_prompt_other

____

5. 45. Have you ever worked UNDERGROUND in a mine?

cancer_subject_baseriskassessmentmining.mine_underground

- Yes
- No
- Do not Know

6. 46. What is the total amount of time you worked UNDERGROUND in the mine?

cancer_subject_baseriskassessmentmining.mine_underground_time

- less than 5 years
- between 5 and 20 years
- more than 20 years

7. 47. When did you last work in a mine?

cancer_subject_baseriskassessmentmining.last_mine

____

Exported from Edc. Revision tag: 1.1.15

Base Risk Assessment: Alcohol

Edc Docstring: BaseRiskAssessmentAlcohol(created, modified, user_created, user_modified, hostname_created, hostname_modified, id, revision, subject_visit_id, report_datetime, alcohol_weekly, amount_drinking)

Instructions: Please complete the questions below.

1. subject visit

cancer_subject_baseriskassessmentalcohol.subject_visit

- dropdown [SubjectVisit]

2. How many days per week do you drink alcohol?

cancer_subject_baseriskassessmentalcohol.alcohol_weekly

____

3. On days you drink, how many drinks do you have (one drink is 300ml of beer/chibuku, 150ml of wine,or 50ml of whiskey/vodka/gin)?

cancer_subject_baseriskassessmentalcohol.amount_drinking

____

Exported from Edc. Revision tag: 1.1.15

Base Risk Assessment: Female

Edc Docstring: BaseRiskAssessmentFemale(created, modified, user_created, user_modified, hostname_created, hostname_modified, id, revision, subject_visit_id, report_datetime, age_period, children, years_breastfed)

Instructions: Please complete the questions below.

1. subject visit

cancer_subject_baseriskassessmentfemale.subject_visit

- dropdown [SubjectVisit]

2. At what age did you start having your menstrual period?

cancer_subject_baseriskassessmentfemale.age_period

____

3. How many children have you given birth to?

cancer_subject_baseriskassessmentfemale.children

____

4. Have you breastfed for a total of at least 1 year? If you have more than 1 child, this includes time spent breast feeding all your children.

cancer_subject_baseriskassessmentfemale.years_breastfed

- Yes
- No

Exported from Edc. Revision tag: 1.1.15

Base Risk Assessment: Chemicals

Edc Docstring: chemical exposure

Instructions: Please complete the questions below.

1. subject visit

cancer_subject_baseriskassessmentchemical.subject_visit

- dropdown [SubjectVisit]

2. Have you ever worked with asbestos without adequate protection?

cancer_subject_baseriskassessmentchemical.asbestos

- Yes
- No
- Do not Know

3. What is the total amount of time you worked with asbestos without protection?

cancer_subject_baseriskassessmentchemical.asbestos_no_protection

- less than 5 years
- between 5 and 20 years
- more than 20 years

4. Have you ever worked with any of these chemical without adequate protection?

cancer_subject_baseriskassessmentchemical.chemicals

- Yes
- No
- Do not Know

Radon, Cadmium, Chromium, Beryllium, Aluminum, Silica, Sulfuric acid, mist, chloromethyl ether, coke (fuel from coal), mustard gas

5. What is the total amount of time you worked with the chemical(s) without protection?

cancer_subject_baseriskassessmentchemical.chemicals_time

- less than 5 years
- between 5 and 20 years
- more than 20 years

6. Have you ever been involved with arsenic smelting (nickel and copper), coal gasification, or iron/steel founding without adequate protection?

cancer_subject_baseriskassessmentchemical.arsenic_smelting

- Yes
- No
- Do not Know

7. What is the total amount of time you worked with the process(es) without protection?

cancer_subject_baseriskassessmentchemical.total_time_no_protection

- less than 5 years
- between 5 and 20 years
- more than 20 years

Exported from Edc. Revision tag: 1.1.15

Base Risk Assessment: Fuel

Edc Docstring: BaseRiskAssessmentFuel(created, modified, user_created, user_modified, hostname_created, hostname_modified, id, revision, subject_visit_id, report_datetime, fuel_20y, fuel_20y_other, cooking, fuel_mm, fuel_mm_other, cooking_mm)

Instructions: Please complete the questions below.

1. subject visit

cancer_subject_baseriskassessmentfuel.subject_visit

- dropdown [SubjectVisit]

2. Over the past 20 years, what type of fuel was used most for cooking/heating in your household?

cancer_subject_baseriskassessmentfuel.fuel_20y

- solid fuels (dung, charcoal, wood, crops, coal)
- kerosene or gas
- electricity
- don't know
- Other, specify

3. ...if "Other", specify

cancer_subject_baseriskassessmentfuel.fuel_20y_other

____

4. Over the past 20 years, was cooking usually done indoors in your household?

cancer_subject_baseriskassessmentfuel.cooking

- Yes
- No
- Do not Know

5. In the past month, what type of fuel was used most for cooking / heating in your household?

cancer_subject_baseriskassessmentfuel.fuel_mm

- solid fuels (dung, charcoal, wood, crops, coal)
- kerosene or gas
- electricity
- don't know
- Other, specify

6. ...if "Other", specify

cancer_subject_baseriskassessmentfuel.fuel_mm_other

____

7. In the past month, was cooking usually done indoors in your household?

cancer_subject_baseriskassessmentfuel.cooking_mm

- Yes
- No
- Do not Know

Exported from Edc. Revision tag: 1.1.15

Base Risk Assessment: Cancer

Edc Docstring: BaseRiskAssessmentCancer(created, modified, user_created, user_modified, hostname_created, hostname_modified, id, revision, subject_visit_id, report_datetime, family_cancer, family_cancer_type, family_cancer_other, had_previous_cancer, previous_cancer, previous_cancer_other)

Instructions: Please complete the questions below.

1. subject visit

cancer_subject_baseriskassessmentcancer.subject_visit

- dropdown [SubjectVisit]

2. Has your son, daughter, brother, sister, or parent ever had cancer?

cancer_subject_baseriskassessmentcancer.family_cancer

- Yes
- No
- Do not Know

3. What kind of cancer did your brother, sister, or parent have?

cancer_subject_baseriskassessmentcancer.family_cancer_type

- Don't know
- Cervical cancer
- Breast cancer
- Esophageal cancer
- Kaposi's sarcoma
- Lymphoma
- Liver cancer
- Eye cancer
- Other or multiple cancers, describe:

4. ...if "Other", specify

cancer_subject_baseriskassessmentcancer.family_cancer_other

____

5. Have you had PREVIOUS cancer, before the current cancer?

cancer_subject_baseriskassessmentcancer.had_previous_cancer

- Yes
- No
- Do not Know

6. What kind of cancer did you have before?

cancer_subject_baseriskassessmentcancer.previous_cancer

- Don't know
- Cervical cancer
- Breast cancer
- Esophageal cancer
- Kaposi's sarcoma
- Lymphoma
- Leukemia
- Wilm's Tumor

- Other or multiple cancers, describe:

7. ...if "Other", specify

cancer_subject_baseriskassessmentcancer.previous_cancer_other

___

Exported from Edc. Revision tag: 1.1.15

Base Risk Assessment: Demographics

Edc Docstring: BaseRiskAssessmentDemo(created, modified, user_created, user_modified, hostname_created, hostname_modified, id, revision, subject_visit_id, report_datetime, marital_status, marital_status_other, race, race_other, ethnic_grp, ethnic_grp_other, community, community_other, district20, setting20, district, setting, education, occupation, occupation_other, money_provide, money_provide_other, money_earned, electricity, toilet, toilet_other, household_people, food_security)

Instructions: Please complete the questions below.

1. subject visit

cancer_subject_baseriskassessmentdemo.subject_visit

- dropdown [SubjectVisit]

2. Marital status:

cancer_subject_baseriskassessmentdemo.marital_status

- Single
- Married
- Cohabiting
- Widowed
- Divorced
- Other, specify

3. ...if "Other", specify

cancer_subject_baseriskassessmentdemo.marital_status_other

___

4. Race:

cancer_subject_baseriskassessmentdemo.race

- Black African
- Caucasian
- Asian
- Other, specify:

5. ...if "Other", specify

cancer_subject_baseriskassessmentdemo.race_other

___

6. Ethnic Group:

cancer_subject_baseriskassessmentdemo.ethnic_grp

- Tswana-Bangwato
- Tswana-Bakwena
- Tswana-Bangwaketsi
- Tswana-Bakgatla
- Tswana-Batawana

- Tswana-Barolong
- Tswana-Bamalete
- Tswana-Batlokwa
- Bakalanga
- Basarwa
- Kgalagadi
- White
- Asian
- Other, specify:

7. ...if "Other", specify

cancer_subject_baseriskassessmentdemo.ethnic_grp_other

____

8. Since 2014, what community have you lived in?

cancer_subject_baseriskassessmentdemo.community

- Bokaa
- Digawana
- Gumare
- Gweta
- Lentsweletau
- Lerala
- Letlhakeng
- Mandunyane
- Mmankgodi
- Mmadinare
- Mmathethe
- Masunga
- Maunatlala
- Mathangwane
- Metsimotlhabe
- Molapowabojang
- Nata
- Nkange
- Oodi
- Otse
- Raikops
- Ramokgonami
- Ranaka
- Sebina
- Sefare
- Sefophe
- Shakawe
- Shoshong
- Tati Siding
- Tsetsebjwe
- Do not want to answer
- Other community

9. ...if "Other", specify

cancer_subject_baseriskassessmentdemo.community_other

____

10. Over the past 20 years, which district have you lived in the most?

cancer_subject_baseriskassessmentdemo.district20

- Central District
- Ghanzi District
- Kgalagadi District
- Kgatleng District
- Kweneng District
- North-East District
- North-West District (includes Chobe/Ngamiland)
- South-East District
- Southern District

11. Over the past 20 years, what best describes the setting you have lived in for most of the time?

cancer_subject_baseriskassessmentdemo.setting20

- Farm/lands
- Village
- City/Town

12. Which district do you live in now?

cancer_subject_baseriskassessmentdemo.district

- Central District
- Ghanzi District
- Kgalagadi District
- Kgatleng District
- Kweneng District
- North-East District
- North-West District (includes Chobe/Ngamiland)
- South-East District
- Southern District

13. What best describes the setting you live in for most of the time now?

cancer_subject_baseriskassessmentdemo.setting

- Farm/lands
- Village
- City/Town

14. Educational level completed:

cancer_subject_baseriskassessmentdemo.education

- None
- Primary
- Junior secondary
- Senior secondary
- Tertiary

15. Occupation:

cancer_subject_baseriskassessmentdemo.occupation

- Housewife
- Salaried (government)
- Salaried (private)
- Domestic work (paid)
- Self-employed
- Student
- Unemployed
- Other, specify:

16. ...if "Other", specify

cancer_subject_baseriskassessmentdemo.occupation_other

____

17. Who provides most of your money:

cancer_subject_baseriskassessmentdemo.money_provide

- Unsure
- You
- Partner or spouse
- Parents
- Other relatives
- Friend
- Other, specify:

18. ...if "Other", specify

cancer_subject_baseriskassessmentdemo.money_provide_other

____

19. How much money do you personally earn?

cancer_subject_baseriskassessmentdemo.money_earned

- None
- <P200/month (< P50/week) * P200-500/month (P50-120/week) * P501-1000/month (P120-230/week) * P1001-2500/month (P230-580/week) * P2501-5000/month (P580-1160/week) * P5001-10000/month (P1160-2330/week) * P10001-20000/month (P2330-4600/week) * P20001-30000/month (P4600-7000/week) * >P30000/month (>P7000/week)

20. Do you have electricity in your house?

cancer_subject_baseriskassessmentdemo.electricity

- Yes
- No

21. Which of the following types of toilet facilities do you most often use at home?

cancer_subject_baseriskassessmentdemo.toilet

- Indoor toilet
- Private latrine for your house/compound
- Shared latrine with other compounds
- No latrine facilities
- Other, specify:

22. ...if "Other", specify

cancer_subject_baseriskassessmentdemo.toilet_other

____

23. How many people, including yourself, stay in your household/compound most of the time?

cancer_subject_baseriskassessmentdemo.household_people

____

24. In the past 4 weeks, did you or any household member have to eat a smaller meal than you felt you needed, or even to skip a meal, becausethere was not enough food?

cancer_subject_baseriskassessmentdemo.food_security

- Never
- Rarely
- Sometimes
- Often
- Patient declined to answer

Exported from Edc. Revision tag: 1.1.15

Base Risk Assessment: Eating

Edc Docstring: eating

Instructions: Please complete the questions below.

1. subject visit

cancer_subject_baseriskassessmenteating.subject_visit

- dropdown [SubjectVisit]

2. Do you eat 5 or more fruit, vegetables, or beans per day?

cancer_subject_baseriskassessmenteating.five_fruit

- Yes
- No
- Do not Know

One serving is one apple, banana or orange, 1 cup of raw leafy vegetable (like spinach or lettuce), 1/2 cup of beans/peas, 1/2 cup of chopped, cooked or canned fruit/vegetable, or 3/4 cup of fruit/vegetable juice. Any fruit, vegetable, or beans qualify.

3. How many meals per week include corn/maize?

cancer_subject_baseriskassessmenteating.meals_weekly

____

4. How many meals per week include sorghum?

cancer_subject_baseriskassessmenteating.meal_sorghum

____

5. How many meals per week include millet?

cancer_subject_baseriskassessmenteating.meal_millet

____

6. How many meals per week include rice?

cancer_subject_baseriskassessmenteating.meal_rice

____

7. How many meals per week include peanuts/groundnuts?

cancer_subject_baseriskassessmenteating.meal_peanuts

____

Exported from Edc. Revision tag: 1.1.15

Cancer Diagnosis

Edc Docstring: CA002

Instructions: Please complete the questions below.

1. subject visit

cancer_subject_cancerdiagnosis.subject_visit

- dropdown [SubjectVisit]

2. GPH ONCO number

cancer_subject_cancerdiagnosis.onco_number

____

3. Pathology number(s)

cancer_subject_cancerdiagnosis.pathology_number

____

4. PM number

cancer_subject_cancerdiagnosis.pm_number

____

5. Has a cancer diagnosis been made?

cancer_subject_cancerdiagnosis.diagnosis

- Yes
- No

6. Category of cancer case:

cancer_subject_cancerdiagnosis.cancer_category

- New Cancer (no treatment for this cancer type for >5 year, or treatment began less than 6 weeks ago
- Relapsed or recurrent cancer (no active treatment for this cancer for >1 year)
- Ongoing treatment (active treatment for this cancer type in past year)

If patient develops a new cancer type (for example, breast cancer after or during treatment for lymphoma) this should be considered a new cancer case.

7. What symptom was most important in prompting patient to seek care leading to diagnosis of cancer?

cancer_subject_cancerdiagnosis.symptom_prompt

- Pain
- Lump/Mass
- Fever
- Cough
- Shortness of Breath
- Bleeding
- Weight loss
- Swelling of leg
- Difficulty swallowing

- Bump/rash on skin or eye
- Other

8. ...if "Other", specify

cancer_subject_cancerdiagnosis.symptom_prompt_other

____

9. When did the patient first notice the symptom (pain, lump, etc.) that led to diagnosis of cancer?

cancer_subject_cancerdiagnosis.symptom_first_noticed

____

10. When did the patient first receive an evaluation by a doctor or nurse for the symptom that led to diagnosis of cancer?

cancer_subject_cancerdiagnosis.first_evaluation

____

11. When did the patient first receive an evaluation by a 'Traditional Doctor or Sangoma' for the symptom that led to diagnosis of cancer?

cancer_subject_cancerdiagnosis.trad_evaluation

____

12. Date of cancer diagnosis

cancer_subject_cancerdiagnosis.date_diagnosed

____

13. Basis of diagnosis

cancer_subject_cancerdiagnosis.diagnosis_basis

- Clinical Only
- Clinical AND Radiology (CT, X-ray, U/S)
- Surgery
- Biochemical/Immunological Test
- Cytology/Haematology
- Histology of Metastasis
- Histology of Primary
- Autopsy with Histology
- Other (including unknown):

14. ...if "Other", specify

cancer_subject_cancerdiagnosis.diagnosis_basis_other

____

15. Diagnosis

cancer_subject_cancerdiagnosis.diagnosis_word

____

In words, metatstatic breast cancer, kaposis of right leg

16. Cancer Site (record ICD topography code)

cancer_subject_cancerdiagnosis.cancer_site

____

17. Clinical and/or Pathologic Diagnosis (record ICD morphology code, M9140/3)

cancer_subject_cancerdiagnosis.clinical_diagnosis

____

18. TNM system- Tumour (T)

cancer_subject_cancerdiagnosis.tumour

- Unknown
- X
- 0
- 1
- 2
- 3
- 4

For Kaposi's record T here, 0 or 1

19. Basis of Tumour (T) assessment

cancer_subject_cancerdiagnosis.tumour_basis

- Unknown
- Clinical
- Pathology

20. TNM system- Lymph Nodes (N)

cancer_subject_cancerdiagnosis.lymph_nodes

- Unknown
- X
- 0
- 1
- 2
- 3

For Kaposi's record I here, 0 or 1

21. Basis of Lymph Node (N) assessment

cancer_subject_cancerdiagnosis.lymph_basis

- Unknown
- Clinical
- Pathology

22. TNM system- Metastasis (M)

cancer_subject_cancerdiagnosis.metastasis

- Unknown
- X
- 0
- 1

For Kaposi's record S here, 0 or 1

23. Basis of Metastasis (M) assessment

cancer_subject_cancerdiagnosis.metastasis_basis

- Unknown
- Clinical
- Pathology

24. Overall cancer stage

cancer_subject_cancerdiagnosis.cancer_stage

- X
- 0
- 1
- 2
- 3
- 4

For lymphomas, report Ann Arbor Stage here. For Kaposi's, report ACTG Stage here.

25. Overall cancer stage modifier

cancer_subject_cancerdiagnosis.cancer_stage_modifier

- Unknown
- No stage modifier
- A
- B
- C
- D

For lymphomas, report Ann Arbor Stage here.For Kaposi's, report 'None'.

26. Are there other results of specialized testing (receptor, cellsurface markers) that should be reported?

cancer_subject_cancerdiagnosis.any_other_results

- Yes
- No

If answered YES, make sure to answer the Specialized Diagnostics form

27. Folder number of stored paper documents

cancer_subject_cancerdiagnosis.paper_documents

____

28. Based the cancer diagnosis or other factors which of the following results be recorded (refer to SOP)?

cancer_subject_cancerdiagnosis.results_to_record select multiple options

- haematology
- chemistry
- tubercolosis
- none

(tick all that apply - REMEMBER to highlight your chosen options before save)

29. ...if "Other", specify

cancer_subject_cancerdiagnosis.results_to_record_other

____

Interform rules (Rule Groups)

1. CancerDiagnosisRuleGroup.results_to_record_4()
   - missing docstring. If True sets *labresultchemistry* and *Lab Result: Haematology* and *Lab Result: Tubercolosis* to 'not_required' otherwise 'new'.
2. CancerDiagnosisRuleGroup.results_to_record_2()
   - missing docstring. If True sets *Lab Result: Chemistry* to 'new' otherwise 'not_required'.
3. CancerDiagnosisRuleGroup.results_to_record_3()
   - missing docstring. If True sets *Lab Result: Tubercolosis* to 'new' otherwise 'not_required'.
4. CancerDiagnosisRuleGroup.results_to_record()
   - missing docstring. If True sets *Lab Result: Haematology* to 'new' otherwise 'not_required'.

Exported from Edc. Revision tag: 1.1.15

Activity and Functioning

Edc Docstring: CA003

Instructions: Please complete the questions below.

1. Today's date

cancer_subject_activityandfunctioning.report_datetime

____

2. subject visit

cancer_subject_activityandfunctioning.subject_visit

- dropdown [SubjectVisit]

3. 1. Overall, how would you rate your health during the PAST 4 WEEKS?

cancer_subject_activityandfunctioning.health_rate

- Excellent
- Very Good
- Good
- Fair
- Poor
- Very Poor

4. 2. During the PAST 4 WEEKS, how much did physical health problems limit your usual physical activities (walking, climbing stairs)?

cancer_subject_activityandfunctioning.health_problems

- Not at all
- Very Little
- Somewhat
- Quite a lot
- Could not do physical activities

5. 3. During the PAST 4 WEEKS, how much difficulty did you have doing your daily work, both at home and away from home, because of your physical health?

cancer_subject_activityandfunctioning.difficulty_work

- None at all
- A little bit
- Some

- Quite a lot
- Could not do daily work

6. 4. How much bodily pain have you had during the PAST 4 WEEKS?

cancer_subject_activityandfunctioning.bodily_pain

- None
- Very mild
- Moderate
- Severe
- Very serere

7. 5. During the PAST 4 WEEKS, how much energy did you have?

cancer_subject_activityandfunctioning.energy

- Very much
- Quite a lot
- Some
- A little
- None

8. 6. During the PAST 4 WEEKS, how much did your physical health or emotional problems limit your usual social activities with family or friends?

cancer_subject_activityandfunctioning.health_probs_limit

- Not at all
- Very little
- Somewhat
- Quite a lot
- Could not do social activities

9. 7. During the PAST 4 WEEKS, how much have you been bothered by emotional problems (such as feeling anxious, depressed or irritable)?

cancer_subject_activityandfunctioning.emotional_probs

- Not at all
- Slightly
- Moderately
- Quite a lot
- Extremely

10. 8. During the past 4 weeks, how much did personal or emotional problems keep you from doing work, school or other daily activities?

cancer_subject_activityandfunctioning.probs_from_work

- Not at all
- Very little
- Somewhat
- Quite a lot
- Could not do daily activities

11. 9. What is the participant perfomance status, determined by study staff with questioning and observation of the participant

cancer_subject_activityandfunctioning.perform_status

- Asymptomatic (Fully active, able to carry on all pre-disease activities without restriction)

- Symptomatic but completely ambulatory (Restricted in physically strenuous activity but ambulatory and able to carry out work of a light or sedentary nature. For example, light housework, office work)
- Symptomatic, <50% in bed during the day (Ambulatory and capable of all self care but unable to carry out any work activities. Up and about more than 50% of waking hours)
- 50% in bed, but not bedbound (Capable of only limited self-care, confined to bed or chair 50% or more of waking hours)
- Bedbound (Completely disabled. Cannot carry on any self-care. Totally confined to bed or chair)
- Death

Exported from Edc. Revision tag: 1.1.15

Oncology Treatment Plan

Edc Docstring: ca004

Instructions: Please complete the questions below.

1. subject visit

cancer_subject_oncologytreatmentplan.subject_visit

- dropdown [SubjectVisit]

2. What is the goal of cancer treatment?

cancer_subject_oncologytreatmentplan.treatment_goal

- Curative
- Palliative
- Unknown

3. Has a treatment plan been determined?

cancer_subject_oncologytreatmentplan.treatment_plan

- Yes
- No

4. Is chemotherapy planned?

cancer_subject_oncologytreatmentplan.chemotherapy

- Yes
- No

5. What was the intent of giving chemotherapy?

cancer_subject_oncologytreatmentplan.chemo_intent

- Standard
- Adjuvant
- Neo-Adjuvant
- Concurrent with radiation

6. Is radiation therapy planned?

cancer_subject_oncologytreatmentplan.radiation_plan

- Yes
- No

7. Is surgical therapy planned?

cancer_subject_oncologytreatmentplan.surgical_plan

- Yes

- No

8. Describe planned operation

    cancer_subject_oncologytreatmentplan.planned_operation

    ____

9. Comments

    cancer_subject_oncologytreatmentplan.comments

    ____

Interform rules (Rule Groups)

1. OncologyTreatmentPlanRuleGroup.radiation_plan()
    - missing docstring. If True sets *Radiation Treatment* to 'new' otherwise 'not_required'.

Exported from Edc. Revision tag: 1.1.15

OTR: Chemotherapy

Edc Docstring: OTRChemo(created, modified, user_created, user_modified, hostname_created, hostname_modified, id, revision, subject_visit_id, report_datetime, chemo_intent, chemo_delays, why_delayed, why_delayed_other, chemo_reduced, why_reduced, why_reduced_other)

Instructions: Please complete the questions below.

1. subject visit

    cancer_subject_otrchemo.subject_visit

- dropdown [SubjectVisit]

2. What was the intent of giving chemotherapy?

    cancer_subject_otrchemo.chemo_intent

- Standard
- Adjuvant
- Neo-Adjuvant
- Concurrent with radiation

3. Were any of the chemotherapy doses/cycles delayed?

    cancer_subject_otrchemo.chemo_delays

- Yes
- No

4. Why were the chemotherapy doses/cycles delayed?

    cancer_subject_otrchemo.why_delayed

- Toxicity - hematologic (anemia, neutropenia, or thromobcytopenia)
- Toxicity - hepatitis (jaundice, increased bilirubin, ALT/AST, etc.)
- Toxicity - renal failure (increased creatinine, swelling, etc)
- Toxicity - other, specify
- Cancer not responding to treatment
- Defaulted visit or lost-to-follow-up
- Outage of medication, supplies, laboratory results
- Clinic too busy to accommodate
- Other, specify

5. ...if "Other", specify

cancer_subject_otrchemo.why_delayed_other

____

6. Were any of the chemotherapy doses (or number of cycles) reduced?

cancer_subject_otrchemo.chemo_reduced

- Yes
- No

7. Why were the chemotherapy doses (or number of cycles) reduced?

cancer_subject_otrchemo.why_reduced

- Toxicity - hematologic (anemia, neutropenia, or thromobcytopenia)
- Toxicity - hepatitis (jaundice, increased bilirubin, ALT/AST, etc.)
- Toxicity - renal failure (increased creatinine, swelling, etc)
- Toxicity - other, specify
- Cancer not responding to treatment
- Defaulted visit or lost-to-follow-up
- Outage of medication, supplies, laboratory results
- Clinic too busy to accommodate
- Dose reduced due to standard protocol (i.e. reduced intensity CHOP)
- Other, specify

8. ...if "Other", specify

cancer_subject_otrchemo.why_reduced_other

____

Exported from Edc. Revision tag: 1.1.15

Chemo Medication Plan

Edc Docstring: ChemoMedPlan(created, modified, user_created, user_modified, hostname_created, hostname_modified, id, revision, drug_code, dose_category, start_date, stop_date, cycle_num, interval, specify_other_med, oncology_treatment_plan_id)

Exported from Edc. Revision tag: 1.1.15

Chemo Medication Record

Edc Docstring: ChemoMedRecord(created, modified, user_created, user_modified, hostname_created, hostname_modified, id, revision, drug_code, dose_category, start_date, stop_date, cycle_num, interval, specify_other_med, otr_chemo_id)

Exported from Edc. Revision tag: 1.1.15

Treatment Response

Edc Docstring: TreatmentResponse(created, modified, user_created, user_modified, hostname_created, hostname_modified, id, revision, subject_visit_id, report_datetime, tx_response_class, tx_response_date, tx_response)

Instructions: Please complete the questions below.

1. subject visit

cancer_subject_treatmentresponse.subject_visit

- dropdown [SubjectVisit]

2. 1. Response to cancer treatment as classified by oncologist / doctor?

cancer_subject_treatmentresponse.tx_response_class

- Progressive disease (tumors are growing or new tumors are appearing)

- Stable disease (no substantial change in size or location of tumors)
- Partial response (at least 50% decrease in tumor size, but less than 100% decrease)
- Complete response (all detectable cancer is gone, 100% decrease)
- Too early after treatment to assess treatment response
- Cannot determine due to pending/missing/unavailable studies (labs, radiology, exam, etc.)
- Not recorded

3. 2. Information used by oncologist / doctor to determine treatment response?

cancer_subject_treatmentresponse.tx_info_determinant select multiple options

- dropdown [InfoDeterminant]

4. 3. Date of assessment of treatment response:

cancer_subject_treatmentresponse.tx_response_date

____

5. 4. Briefly describe response to treatment and information used to judge treatment response:

cancer_subject_treatmentresponse.tx_response

____

Exported from Edc. Revision tag: 1.1.15

Referral

Edc Docstring: CA011

Exported from Edc. Revision tag: 1.1.15

Locator

Edc Docstring: Locator(created, modified, user_created, user_modified, hostname_created, hostname_modified, id, revision, registered_subject_id, report_datetime, date_signed, mail_address, home_visit_permission, physical_address, may_follow_up, may_sms_follow_up, subject_cell, subject_cell_alt, subject_phone, subject_phone_alt, may_call_work, subject_work_place, subject_work_phone, may_contact_someone, contact_name, contact_rel, contact_physical_address, contact_cell, contact_phone, subject_visit_id, alt_contact_cell_number, has_alt_contact, alt_contact_name, alt_contact_rel, alt_contact_cell, other_alt_contact_cell, alt_contact_tel, local_clinic, home_village)

Instructions: Please complete the questions below.

1. subject visit

cancer_subject_locator.subject_visit

- dropdown [SubjectVisit]

2. Date Locator Form signed

cancer_subject_locator.date_signed

____

3. Mailing address

cancer_subject_locator.mail_address

____

(Encryption: aes local)

4. Has the participant given his/her permission for study staff to make home visits for follow-up purposes during the study?

cancer_subject_locator.home_visit_permission

- Yes
- No

5. Physical address with detailed description

cancer_subject_locator.physical_address

____

(Encryption: aes local)

6. Has the participant given his/her permission for study staff to call her for follow-up purposes during the study?

cancer_subject_locator.may_follow_up

- Yes
- No

7. Cell number

cancer_subject_locator.subject_cell

____

(Encryption: rsa local)

8. Cell number (alternate)

cancer_subject_locator.subject_cell_alt

____

(Encryption: rsa local)

9. Telephone

cancer_subject_locator.subject_phone

____

(Encryption: rsa local)

10. Telephone (alternate)

cancer_subject_locator.subject_phone_alt

____

(Encryption: rsa local)

11. Has the participant given his/her permission for study staff to contact anyone else for follow-up purposes during the study?

cancer_subject_locator.may_contact_someone

- Yes
- No

For example a partner, spouse, family member, neighbour ...

12. Full names of the contact person

cancer_subject_locator.contact_name

____

(Encryption: rsa local)

13. Relationship to participant

cancer_subject_locator.contact_rel

____

(Encryption: rsa local)

14. Cell number

cancer_subject_locator.contact_cell

____

(Encryption: rsa local)

15. Cell number (alternate)

cancer_subject_locator.alt_contact_cell_number

____

(Encryption: rsa local)

16. Telephone number

cancer_subject_locator.contact_phone

____

(Encryption: rsa local)

17. If we are unable to contact the person indicated above, is there another individual (including next of kin) with whom the study team can get in contact with?

cancer_subject_locator.has_alt_contact

- Yes
- No

18. Full Name of the responsible person

cancer_subject_locator.alt_contact_name

____

include firstname and surname (Encryption: rsa local)

19. Relationship to participant

cancer_subject_locator.alt_contact_rel

____

(Encryption: rsa local)

20. Cell number

cancer_subject_locator.alt_contact_cell

____

(Encryption: rsa local)

21. Cell number (alternate)

cancer_subject_locator.other_alt_contact_cell

____

(Encryption: rsa local)

22. Telephone number

cancer_subject_locator.alt_contact_tel

____

(Encryption: rsa local)

23. Has the participant given his/her permission for study staff to contact her at work for follow up purposes during the study?

cancer_subject_locator.may_call_work

- Yes
- No
- Doesnt Work

24. Name and location of work place

cancer_subject_locator.subject_work_place

____

(Encryption: aes local)

25. Work telephone number

cancer_subject_locator.subject_work_phone

____

(Encryption: rsa local)

26. Where is your home village?

cancer_subject_locator.home_village

____

27. When you stay in the village, what clinic/health post do you normally go to?

cancer_subject_locator.local_clinic

____

Exported from Edc. Revision tag: 1.1.15

Haart Record

Edc Docstring: MC034

Instructions: Please complete the questions below.

1. subject visit

cancer_subject_haartrecord.subject_visit

- dropdown [SubjectVisit]

2. What is the status of the participant's antiretroviral treatment (HAART)?

cancer_subject_haartrecord.haart_status

- Never started HAART "(skip to Question 4)"
- Follow-up visit, no modifications since last visit made to HAART treatment "(skip to Question 4)"
- Enrollment visit, patient has taken or is taking HAART "(go to Question 3, record all current and past HAART medications)"
- Change in at least one antiretroviral medication (dose modification, discontinuation, temporary hold, change of medication) "(go to Question 3)"

3. Comments

cancer_subject_haartrecord.comments

____

Exported from Edc. Revision tag: 1.1.15

Haart Medication

Edc Docstring: HaartMedRecord(created, modified, user_created, user_modified, hostname_created, hostname_modified, id, revision, drug_name, mod_reason, arv_reason, start_date, stop_date, haart_record_id)

Exported from Edc. Revision tag: 1.1.15

Lab Result

Edc Docstring: CA005 Lab Result

Instructions: Please complete the questions below.

1. subject visit

cancer_subject_labresult.subject_visit

- dropdown [SubjectVisit]

2. Are there any new HIV TEST results that have not been previously reported?

cancer_subject_labresult.has_hiv_result

- Yes
- No

3. Are there any new CD4 CELL COUNT results that have not been previously reported?

cancer_subject_labresult.has_cd4

- Yes
- No

4. Are there any new HIV VIRAL LOAD results that have not been previously reported?

cancer_subject_labresult.has_vl

- Yes
- No

5. Are there any new HAEMOTOLOGY results that have not been previously reported?

cancer_subject_labresult.has_haem

- Yes
- No

6. Are there any new CHEMISTRY results that have not been previously reported?

cancer_subject_labresult.has_chem

- Yes
- No

7. Are there any new OTHER ABNORMAL laboratory results not been previously reported that have changed or delayed planned treatment?

cancer_subject_labresult.has_other_abnormal

- Yes
- No

8. ...if "Other", specify

cancer_subject_labresult.other_abnormal

____

9. Are there any tuberculosis diagnostic tests that have not been previously reported?

cancer_subject_labresult.tb_tests

- Yes
- No

10. ...if "Other", specify

cancer_subject_labresult.tb_prompt_other

____

Exported from Edc. Revision tag: 1.1.15

Lab Result: HIV

Edc Docstring: LabResultHiv(created, modified, user_created, user_modified, hostname_created, hostname_modified, id, revision, subject_visit_id, report_datetime, test_date, test_result)

Instructions: Please complete the questions below.

1. subject visit

cancer_subject_labresulthiv.subject_visit

- dropdown [SubjectVisit]

2. 2. Date of HIV test

cancer_subject_labresulthiv.test_date

____

3. 3. HIV test result

cancer_subject_labresulthiv.test_result

- Reactive
- Non-Reactive

Exported from Edc. Revision tag: 1.1.15

Lab Result: CD4

Edc Docstring: LabResultCd4(created, modified, user_created, user_modified, hostname_created, hostname_modified, id, revision, subject_visit_id, report_datetime, cd4_drawn_date, cd4_result)

Instructions: Please complete the questions below.

1. subject visit

cancer_subject_labresultcd4.subject_visit

- dropdown [SubjectVisit]

2. 5. Date of CD4 cell count

cancer_subject_labresultcd4.cd4_drawn_date

____

3. 6. CD4 cell count result

cancer_subject_labresultcd4.cd4_result

____

Exported from Edc. Revision tag: 1.1.15

Lab Result: Viral Load

Edc Docstring: LabResultViralload(created, modified, user_created, user_modified, hostname_created, hostname_modified, id, revision, subject_visit_id, report_datetime, vl_drawn_date, vl_result)

Instructions: Please complete the questions below.

1. subject visit

cancer_subject_labresultviralload.subject_visit

- dropdown [SubjectVisit]

2. Today's date

cancer_subject_labresultviralload.report_datetime

____

3. 8. Date of HIV viral load

cancer_subject_labresultviralload.vl_drawn_date

____

4. 9. HIV viral load result

cancer_subject_labresultviralload.vl_result

____

Exported from Edc. Revision tag: 1.1.15

Lab Result: Haematology

Edc Docstring: LabResultHaematology(created, modified, user_created, user_modified, hostname_created, hostname_modified, id, revision, subject_visit_id, report_datetime, haem_drawn_date, hgb, mcv, wbc_count, anc_count, platelet, comments)

Instructions: Please complete the questions below.

1. subject visit

cancer_subject_labresulthaematology.subject_visit

- dropdown [SubjectVisit]

2. Date of haematology specimen draw

cancer_subject_labresulthaematology.haem_drawn_date

____

3. Haemoglobin

cancer_subject_labresulthaematology.hgb

____

mg/dL

4. Mean corpuscular volume (MCV):

cancer_subject_labresulthaematology.mcv

____

microL

5. White blood cell (WBC) count :

cancer_subject_labresulthaematology.wbc_count

____

cells/microL

6. Absolute neutrophil count (ANC) :

    cancer_subject_labresulthaematology.anc_count

    ____

cells/microL

7. Platelet count:

    cancer_subject_labresulthaematology.platelet

    ____

cells/microL

8. Comments:

    cancer_subject_labresulthaematology.comments

    ____

if other data not recorded, explain why

Exported from Edc. Revision tag: 1.1.15

Lab Result: Chemistry

Edc Docstring: LabResultChemistry(created, modified, user_created, user_modified, hostname_created, hostname_modified, id, revision, subject_visit_id, report_datetime, chem_drawn_date, alanine, aspartate, bilirubin, creatinine, lactate, comments)

Instructions: Please complete the questions below.

1. subject visit

    cancer_subject_labresultchemistry.subject_visit

- dropdown [SubjectVisit]

2. Date of chemistry specimen draw:

    cancer_subject_labresultchemistry.chem_drawn_date

    ____

3. Alanine aminotransferase (ALT or SGPT):

    cancer_subject_labresultchemistry.alanine

    ____

U/L

4. Aspartate aminotransferase (AST or SGOT):

    cancer_subject_labresultchemistry.aspartate

    ____

U/L

5. Bilirubin:

    cancer_subject_labresultchemistry.bilirubin

    ____

mg/dL

6. Creatinine:

    cancer_subject_labresultchemistry.creatinine

    ____

umol/L

7. Lactate Dehydrogenase (LDH):

cancer_subject_labresultchemistry.lactate

____

IU/L

8. Comments:

cancer_subject_labresultchemistry.comments

____

if other data not recorded, explain why

Exported from Edc. Revision tag: 1.1.15

Lab Result: Tubercolosis

Edc Docstring: LabResultTb(created, modified, user_created, user_modified, hostname_created, hostname_modified, id, revision, subject_visit_id, report_datetime, tb_description, tb_treatment, tb_treatment_start)

Instructions: Please complete the questions below.

1. subject visit

cancer_subject_labresulttb.subject_visit

- dropdown [SubjectVisit]

2. Describe tubercolosis diagnostic test results (record test, date, result and units)

cancer_subject_labresulttb.tb_description

____

3. Is participant being treated for tuberculosis now?

cancer_subject_labresulttb.tb_treatment

- No
- Yes, isoniazid preventative therapy (IPT)
- Yes, combination anti-tuberculosis treatment (ATT)

4. When did the participant's treatment for tuberculosis begin?

cancer_subject_labresulttb.tb_treatment_start

____

Exported from Edc. Revision tag: 1.1.15

Lab Result: Height & Weight

Edc Docstring: CA005 Lab Result : Height Weight Cough patient information to " be filled by Recruiter

Instructions: Please complete the questions below.

1. subject visit

cancer_subject_labresultheightweight.subject_visit

- dropdown [SubjectVisit]

2. Weight

cancer_subject_labresultheightweight.weight

____

kg

3. Height:

cancer_subject_labresultheightweight.height

____

cm

4. Does the participant have cough (>2 weeks) OR weight loss OR drenching night sweats (need to change bed clothes/sheets)?

cancer_subject_labresultheightweight.cough2weeks

- Yes
- No

Exported from Edc. Revision tag: 1.1.15

Oncology Treatment Record

Edc Docstring: CA007

Instructions: Note to Interviewer: If any of the answers below are yes, make arrangements to obtain records or review records over the phone

1. subject visit

cancer_subject_oncologytreatmentrecord.subject_visit

- dropdown [SubjectVisit]

2. Has the patient COMPLETED chemotherapy?

cancer_subject_oncologytreatmentrecord.chemo_received

- Yes
- No

3. Did the patient COMPLETE radiation therapy?

cancer_subject_oncologytreatmentrecord.radiation_received

- Yes
- No

4. Did patient COMPLETE surgical therapy?

cancer_subject_oncologytreatmentrecord.surgical_therapy

- Yes
- No

5. Comments:

cancer_subject_oncologytreatmentrecord.comments

____

Interform rules (Rule Groups)

1. OncologyTreatmentRecordRuleGroup.radiation_received()
   - missing docstring. If True sets *Radiation Treatment* to 'new' otherwise 'not_required'.
2. OncologyTreatmentRecordRuleGroup.surgical_therapy()
   - Returns True if surgical_therapy equals No. If True sets *OTR: Surgical* to 'not_required' otherwise 'new'.
3. OncologyTreatmentRecordRuleGroup.chemo_received()
   - Returns True if chemo_received equals No. If True sets *OTR: Chemotherapy* to 'not_required' otherwise 'new'.

Exported from Edc. Revision tag: 1.1.15

OTR: Radiation

Edc Docstring: OTRRadiation(created, modified, user_created, user_modified, hostname_created, hostname_modified, id, revision, subject_visit_id, report_datetime, radiation_details)

Instructions: Review the recorded cancer type and stage information recorded and consider updating 'Cancer Diagnosis' form accordingly.

1. subject visit

cancer_subject_otrradiation.subject_visit

- dropdown [SubjectVisit]

2. Are there radiation details available?

cancer_subject_otrradiation.radiation_details

- Yes
- No

Exported from Edc. Revision tag: 1.1.15

OTR: Surgical

Edc Docstring: OTRSurgical(created, modified, user_created, user_modified, hostname_created, hostname_modified, id, revision, subject_visit_id, report_datetime, operation_performed, date_operation)

Instructions: Please complete the questions below.

1. subject visit

cancer_subject_otrsurgical.subject_visit

- dropdown [SubjectVisit]

2. What operation was performed?:

cancer_subject_otrsurgical.operation_performed

____

3. Date of operation?

cancer_subject_otrsurgical.date_operation

____

Exported from Edc. Revision tag: 1.1.15

Baseline HIV History

Edc Docstring: CA006

Instructions: Please complete the questions below.

1. subject visit

cancer_subject_baselinehivhistory.subject_visit

- dropdown [SubjectVisit]

2. Has the participant been previously tested for HIV?

cancer_subject_baselinehivhistory.has_hiv_result

- Yes
- No
- Do not Know

3. Has patient ever had any WHO stage 3 or 4 illnesses?

cancer_subject_baselinehivhistory.had_who_illnesses

- Yes
- No

Refer to WHO classification document. DO NOT include the current cancer diagnosis.

4. Are 'CD4' results available?

cancer_subject_baselinehivhistory.has_cd4

- Yes
- No

5. What is the value of the most recent CD4 result (closest to time of cancer diagnosis)

cancer_subject_baselinehivhistory.cd4_result

____

4-digit number field

6. Date of recent CD4?

cancer_subject_baselinehivhistory.cd4_drawn_date

____

7. Is a CD4 result lower than the most recent CD4 result available?

cancer_subject_baselinehivhistory.has_prior_cd4

- Yes
- No

8. What is the value of the lowest CD4 result recorded

cancer_subject_baselinehivhistory.nadir_cd4

____

4-digit number field

9. Date of lowest CD4

cancer_subject_baselinehivhistory.nadir_cd4_drawn_date

____

10. Are 'VIRAL LOAD' results available?

cancer_subject_baselinehivhistory.has_vl

- Yes
- No

11. HIV viral load result

cancer_subject_baselinehivhistory.vl_result

____

12. Date of HIV viral load

cancer_subject_baselinehivhistory.vl_drawn_date

____

Interform rules (Rule Groups)

1. BaselineHIVHistoryRuleGroup.had_who_illnesses()
   - Returns True if had_who_illnesses equals No. If True sets *BHH: WHO illness* to 'not_required' otherwise 'new'.
2. BaselineHIVHistoryRuleGroup.has_hiv_result()
   - Returns True if has_hiv_result equals No OR has_hiv_result equals Dont_know. If True sets *BHH: HIV Test* to 'not_required' otherwise 'new'.

Exported from Edc. Revision tag: 1.1.15

BHH: HIV Test

Edc Docstring: BHHHivTest(created, modified, user_created, user_modified, hostname_created, hostname_modified, id, revision, subject_visit_id, report_datetime, hiv_drawn_date, hiv_testdate_est, hiv_result)

Instructions: Please complete the questions below.

1. subject visit

cancer_subject_bhhhivtest.subject_visit

- dropdown [SubjectVisit]

2. Date of most recent HIV test:

cancer_subject_bhhhivtest.hiv_drawn_date

____

3. Is the HIV test date estimated?

cancer_subject_bhhhivtest.hiv_testdate_est

- Yes
- No

4. Result of most recent HIV test:

cancer_subject_bhhhivtest.hiv_result

- Reactive (positive)
- Non-Reactive (negative)
- Don't Know (didn't receive result, forgot, etc)

If last HIV test negative (or Don't Know) and more than six months ago, perform HIV testing unless patient refuses.

Interform rules (Rule Groups)

1. BHHHivTestRuleGroup.hiv_result()
   - Returns True if hiv_result equals POS. If True sets *Haart Record* to 'new' otherwise 'not_required'.
2. BHHHivTestRuleGroup.also_hiv_result()
   - Returns True if hiv_result equals NEG OR hiv_result equals UKN. If True sets *Haart Record* to 'not_required' otherwise 'new'.

Exported from Edc. Revision tag: 1.1.15

BHH: WHO illness

Edc Docstring: BHHWhoIllness(created, modified, user_created, user_modified, hostname_created, hostname_modified, id, revision, subject_visit_id, report_datetime, who_illness_other, who_illness_date)

Instructions: Please complete the questions below.

1. subject visit

cancer_subject_bhhwhoillness.subject_visit

- dropdown [SubjectVisit]

2. Date of most recent WHO stage 3 or 4 illness:

cancer_subject_bhhwhoillness.who_illness_date

____

DO NOT include the current cancer diagnosis.

3. What WHO stage 3 or 4 illnesses the patient had:

cancer_subject_bhhwhoillness.who_illness select multiple options

- Wasting
- Tuberculosis
- Kaposi's sarcoma
- Kidney failure
- Cryptococcal meningitis
- Severe bacterial infections
- Other, specify

Tick all that apply. DO NOT include current cancer diagnosis

4. ...if "Other", specify

cancer_subject_bhhwhoillness.who_illness_other

____

Exported from Edc. Revision tag: 1.1.15

BHH: CD4

Edc Docstring: BHHCd4(created, modified, user_created, user_modified, hostname_created, hostname_modified, id, revision, subject_visit_id, report_datetime, nadir_cd4, nadir_cd4_drawn_date)

Instructions: Please complete the questions below.

1. subject visit

cancer_subject_bhhcd4.subject_visit

- dropdown [SubjectVisit]

2. 8. What is the value of the lowest 'CD4' test recorded?

cancer_subject_bhhcd4.nadir_cd4

____

If current (most recent) CD4 is lowest recorded, record again here.

3. 9. Date 'CD4' test was run:

cancer_subject_bhhcd4.nadir_cd4_drawn_date

____

Exported from Edc. Revision tag: 1.1.15

Symptoms and Testing

Edc Docstring: CA015

Instructions: Please complete the questions below.

1. Today's date

cancer_subject_symptomsandtesting.report_datetime

____

2. subject visit

cancer_subject_symptomsandtesting.subject_visit

- dropdown [SubjectVisit]

3. What symptom was most important in prompting you to seek care leading to a diagnosis of cancer (ie pain, lump, fever, bleeding, etc)?

cancer_subject_symptomsandtesting.symptom_prompt

____

4. When did you first notice the symptom that led to a diagnosis of cancer?

cancer_subject_symptomsandtesting.symptom_date

____

5. When did you first see a medical doctor for the symptom?

cancer_subject_symptomsandtesting.medical_doctor_date

____

6. When did you first see a traditional doctor for the symptom?

cancer_subject_symptomsandtesting.trad_doctor_date

____

7. In which facility was this symptom first presented?

cancer_subject_symptomsandtesting.facility_first_seen

____

provide name of clinic if facility code is unknown or is 00-0-00

8. Please provide name of clinic

cancer_subject_symptomsandtesting.facility_first_seen_other

____

9. Have you ever been tested for HIV?

cancer_subject_symptomsandtesting.hiv_tested

- Yes
- No
- Refused to answer

10. What was the most recent HIV test result?

cancer_subject_symptomsandtesting.hiv_test_result

- Positive
- Negative
- Indeterminate
- Refused to disclose

11. When was your first positive HIV test?

cancer_subject_symptomsandtesting.pos_date

____

12. When was your last negative HIV test?

cancer_subject_symptomsandtesting.neg_date

____

if 'within the last 6 months' END form

13. HIV test result

cancer_subject_symptomsandtesting.hiv_result

- Positive (both rapid tests)
- Negative (both rapid tests)
- Indeterminate (different results on rapid tests)
- Result pending (sent to lab waiting for result)
- Patient refuses HIV testing today

Provide appropriate post-test counselling and referral to care. If indeterminate, send patient to the lab for re-testing and ELISA

14. Have you ever taken anti-retroviral therapy or HAART?

cancer_subject_symptomsandtesting.arv_art_therapy

- Yes
- No
- Refused to answer

if 'NO' END form

15. When did you start antiretroviral therapy, or HAART

cancer_subject_symptomsandtesting.arv_art_start_date

____

16. Are you taking antiretroviral therapy or HAART now?

cancer_subject_symptomsandtesting.arv_art_now

- Yes
- No
- Refused to answer

if 'Yes' END form

17. When did you most recently stop antiretroviral therapy, or HAART?

cancer_subject_symptomsandtesting.art_art_stop_date

____

Interform rules (Rule Groups)

1. SymptomsTestingRuleGroup.hiv_test_result()
   - Returns True if hiv_result ne Pos OR hiv_test_result ne POS. If True sets *bhhhivtest* and *haartrecord* and *Baseline HIV History* and *BHH: WHO illness* to 'not_required' otherwise 'new'.

Exported from Edc. Revision tag: 1.1.15

Radiation Treatment

Edc Docstring: RadiationTreatment(created, modified, user_created, user_modified, hostname_created, hostname_modified, id, revision, subject_visit_id, report_datetime, treatment_start_date, treatment_end_date, tumour_stages, lymph_stages, metastasis_stages, overall_stages, stage_modifier, treatment_itent, treatment_relationship, side_effects_other, response, response_other, any_missed_doses, if_doses_missed, if_doses_missed_other, any_doses_delayed, if_doses_delayed, if_doses_delayed_other, first_course_radiation, comments)

Instructions: Please complete the questions below.

1. subject visit

cancer_subject_radiationtreatment.subject_visit

- dropdown [SubjectVisit]

2. Treatment start date

cancer_subject_radiationtreatment.treatment_start_date

____

3. Treatment end date

cancer_subject_radiationtreatment.treatment_end_date

____

4. TNM system- Tumour (T) stage recorded in radiation records:

cancer_subject_radiationtreatment.tumour_stages

- X
- 0
- 1
- 2
- 3
- 4

For Kaposi's record T here, 0 or 1

5. TNM system- Lymph Nodes (N) stage recorded in radiation records:

cancer_subject_radiationtreatment.lymph_stages

- X
- 0
- 1
- 2
- 3
- 4

For Kaposi's record I here, 0 or 1

6. TNM system- Metastasis (M) stage recorded in radiation records:

cancer_subject_radiationtreatment.metastasis_stages

- X
- 0
- 1
- 2
- 3
- 4

For Kaposi's record S here, 0 or 1

7. Overall cancer stage

cancer_subject_radiationtreatment.overall_stages

- X
- 0
- 1
- 2
- 3
- 4

For lymphomas, report Ann Arbor Stage here. For Kaposi's, report ACTG Stage here.

8. Overall cancer stage modifier

cancer_subject_radiationtreatment.stage_modifier

- X
- A
- B
- C
- D

For lymphomas, report Ann Arbor Stage here.For Kaposi's, report 'None'.

9. Treatment intent

cancer_subject_radiationtreatment.treatment_itent

- Unknown
- Curative
- Palliative

10. Relationship to other treatment modalities

cancer_subject_radiationtreatment.treatment_relationship

- Unknown
- No other treatment modalities

- Concurrent chemotherapy
- Adjuvant after surgery
- Adjuvant after chemotherapy
- Adjuvant after surgery and chemotherapy
- Neoadjuvant before Chemotherapy
- Neoadjuvant before Surgery
- Other, specify

11. Side Effects

    cancer_subject_radiationtreatment.side_effects select multiple options

- UNK
- hyperpigmentation
- vaginal stenosis
- diarrhea, proctitis
- moist desquamation
- fibrosis
- Other, specify
- None
- Escoriation
- Dry Desquamation
- Lymphoedema

(tick all that apply)

12. ...if "Other", specify

cancer_subject_radiationtreatment.side_effects_other

____

13. Response to Treatment

    cancer_subject_radiationtreatment.response

- Unknown
- Complete
- Almost Complete
- Residual Tumor
- Poor response
- Good palliation
- Modest Palliation
- Poor Palliation
- Other, specify

14. ...if "Other", specify

cancer_subject_radiationtreatment.response_other

____

15. Were any doses missed

    cancer_subject_radiationtreatment.any_missed_doses

- Yes
- No
- Unknown

16. If yes, why were treatments missed?

cancer_subject_radiationtreatment.if_doses_missed

- Toxicity- hematologic (anemia, neutropenia, or low plts),
- Toxicity-skin (dermatitis, mucositis),
- Cancer not responding to treatment
- Defaulted visit or lost to follow-up
- Machine down-time or repair
- clinic too busy to accommodate
- lack of transportation to facility
- Other, specify

17. ...if "Other", specify

cancer_subject_radiationtreatment.if_doses_missed_other

____

18. Were any doses delayed

cancer_subject_radiationtreatment.any_doses_delayed

- Yes
- No
- Unknown

19. If yes, why were treatments delayed?

cancer_subject_radiationtreatment.if_doses_delayed

- Toxicity- hematologic (anemia, neutropenia, or low plts),
- Toxicity-skin (dermatitis, mucositis),
- Cancer not responding to treatment
- Defaulted visit or lost to follow-up
- Machine down-time or repair
- clinic too busy to accommodate
- lack of transportation to facility
- Other, specify

20. ...if "Other", specify

cancer_subject_radiationtreatment.if_doses_delayed_other

____

21. Was this the first course of radiation

cancer_subject_radiationtreatment.first_course_radiation

- Yes
- No
- Unknown

22. Comments

cancer_subject_radiationtreatment.comments

____

Exported from Edc. Revision tag: 1.1.15

Radiation Treatment Record

Edc Docstring: RadiationTreatmentRecord(created, modified, user_created, user_modified, hostname_created, hostname_modified, id, revision, treatment_name, start_date, end_date, dose_delivered, dose_described, fractions, dose_per_fraction, radiation_technique, radiation_technique_other, modality, brachy_length, brachy_type, radiation_treatment_id)

Exported from Edc. Revision tag: 1.1.15

Oncology Treatment Completed

Edc Docstring: NEW form on system upgrade

Instructions: Please complete the questions below.

1. subject visit

cancer_subject_oncologytreatmentcompleted.subject_visit

- dropdown [SubjectVisit]

2. Has the patient had chemotherapy?

cancer_subject_oncologytreatmentcompleted.patient_had_chemo

- Yes
- No
- Not Sure

3. Has the patient had radiation therapy?

cancer_subject_oncologytreatmentcompleted.patient_had_radiation

- Yes
- No
- Not Sure

4. Has the patient had surgery?

cancer_subject_oncologytreatmentcompleted.patient_had_surgery

- Yes
- No
- Not Sure

5. Describe any details of the treatment?

cancer_subject_oncologytreatmentcompleted.treatment_detail

____

(dates, cycles, drugs, order of treatment, etc)

6. Where is the patient being followed?

cancer_subject_oncologytreatmentcompleted.patient_follow_up

- Princess Marina Hospital
- Nyangabgwe Referral Hospital
- Serowe
- Maun
- Other, specify

7. ...if "Other", specify

cancer_subject_oncologytreatmentcompleted.patient_follow_up_other

____

Interform rules (Rule Groups)

1. OncologyTreatmentCompletedRuleGroup.patient_had_radiation()
   - Returns True if patient_had_radiation equals Yes. If True sets *Radiation Treatment* to 'new' otherwise 'not_required'.
2. OncologyTreatmentCompletedRuleGroup.patient_had_surgery()
   - Returns True if patient_had_surgery equals Yes. If True sets *OTR: Surgical* to 'new' otherwise 'not_required'.
3. OncologyTreatmentCompletedRuleGroup.patient_had_chemo()
   - Returns True if patient_had_chemo equals Yes. If True sets *OTR: Chemotherapy* to 'new' otherwise 'not_required'.

Exported from Edc. Revision tag: 1.1.15

Current Symptoms

Edc Docstring: NEW form on system upgrade

Instructions: Please complete the questions below.

1. subject visit

cancer_subject_currentsymptoms.subject_visit

- dropdown [SubjectVisit]

2. Does the patient have any symptoms they are worried about?

cancer_subject_currentsymptoms.any_worry

- Yes
- No
- Not Sure

3. If so, describe their symptom

cancer_subject_currentsymptoms.symptom_desc

____

4. What has the patient tried to do about the symptom?

cancer_subject_currentsymptoms.patient_own_remedy

____

5. Severity

cancer_subject_currentsymptoms.severity

- Not Applicable
- MILD symptoms causing no or minimal interference with usual social and functional activities with intervention not indicated.
- MODERATE symptoms causing greater than minimal interference with usual social and functional activities with intervention indicated.
- SEVERE symptoms causing inability to perform usual social and functional activities with intervention or hospitalization indicated.
- POTENTIALLY LIFE-THREATENING symptoms causing inability to perform basic self-care functions with intervention indicated to prevent permanent impairment, persistent disability, or death.

If you determine that participant could have Grade 4 illness please assist them to as best as possible by immediately informing the Oncology clinicians and the Study Coordinator.

6. What did the RA do to help?

cancer_subject_currentsymptoms.ra_advice

---

7. Outcome or Update

cancer_subject_currentsymptoms.outcome_update

Exported from Edc. Revision tag: 1.1.15

Article Sources and Contributors

BHP045 Edc Exported Form Text: Cancer Subject Package 20160308 *Source*: http://wiki.bhp.org.bw/index.php?oldid=62243 *Contributors*: Onep
